# Intestinal monocarboxylate transporter 1 mediates lactate transport in the gut and regulates metabolic homeostasis of mouse in a sex-dimorphic pattern

**DOI:** 10.1093/lifemeta/load041

**Published:** 2023-11-06

**Authors:** Shuo Wang, Lingling Zhang, Jingyu Zhao, Meijuan Bai, Yijun Lin, Qianqian Chu, Jue Gong, Ju Qiu, Yan Chen

**Affiliations:** CAS Key Laboratory of Nutrition, Metabolism and Food Safety, Shanghai Institute of Nutrition and Health, University of Chinese Academy of Sciences, Chinese Academy of Sciences, Shanghai 200031, China; CAS Key Laboratory of Nutrition, Metabolism and Food Safety, Shanghai Institute of Nutrition and Health, University of Chinese Academy of Sciences, Chinese Academy of Sciences, Shanghai 200031, China; CAS Key Laboratory of Nutrition, Metabolism and Food Safety, Shanghai Institute of Nutrition and Health, University of Chinese Academy of Sciences, Chinese Academy of Sciences, Shanghai 200031, China; CAS Key Laboratory of Nutrition, Metabolism and Food Safety, Shanghai Institute of Nutrition and Health, University of Chinese Academy of Sciences, Chinese Academy of Sciences, Shanghai 200031, China; CAS Key Laboratory of Nutrition, Metabolism and Food Safety, Shanghai Institute of Nutrition and Health, University of Chinese Academy of Sciences, Chinese Academy of Sciences, Shanghai 200031, China; CAS Key Laboratory of Nutrition, Metabolism and Food Safety, Shanghai Institute of Nutrition and Health, University of Chinese Academy of Sciences, Chinese Academy of Sciences, Shanghai 200031, China; CAS Key Laboratory of Tissue Microenvironment and Tumor, Shanghai Institute of Nutrition and Health, University of Chinese Academy of Sciences, Chinese Academy of Sciences, Shanghai 200031, China; CAS Key Laboratory of Tissue Microenvironment and Tumor, Shanghai Institute of Nutrition and Health, University of Chinese Academy of Sciences, Chinese Academy of Sciences, Shanghai 200031, China; CAS Key Laboratory of Nutrition, Metabolism and Food Safety, Shanghai Institute of Nutrition and Health, University of Chinese Academy of Sciences, Chinese Academy of Sciences, Shanghai 200031, China

**Keywords:** MCT1, intestine, lactate, short-chain fatty acids, insulin resistance, obesity

## Abstract

The monocarboxylate transporter 1 (MCT1), encoded by gene *Slc16a1*, is a proton-coupled transporter for lactate and other monocarboxylates. MCT1-mediated lactate transport was recently found to regulate various biological functions. However, how MCT1 and lactate in the intestine modulate the physiology and pathophysiology of the body is unclear. In this study, we generated a mouse model with specific deletion of *Slc16a1* in the intestinal epithelium (*Slc16a1*^IKO^ mice) and investigated the functions of MCT1 in the gut. When fed a high-fat diet, *Slc16a1*^IKO^ male mice had improvement in glucose tolerance and insulin sensitivity, while *Slc16a1*^IKO^ female mice only had increased adiposity. Deficiency of intestinal MCT1 in male mice was associated with downregulation of pro-inflammatory pathways, together with decreased circulating levels of inflammatory cytokines including tumor necrosis factor alpha (TNFα) and C–C motif chemokine ligand 2 (CCL2). Lactate had a stimulatory effect on pro-inflammatory macrophages *in vitro*. The number of intestinal macrophages was reduced in *Slc16a1*^IKO^ male mice *in vivo*. Intestinal deletion of *Slc16a1* in male mice reduced interstitial lactate level in the intestine. In addition, treatment of male mice with estrogen lowered interstitial lactate level in the intestine and abolished the difference in glucose homeostasis between *Slc16a1*^IKO^ and wild-type mice. Deficiency of intestinal MCT1 also blocked the transport of lactate and short-chain fatty acids from the intestine to the portal vein. The effect of *Slc16a1* deletion on glucose homeostasis in male mice was partly mediated by alterations in gut microbiota. In conclusion, our work reveals that intestinal MCT1 regulates glucose homeostasis in a sex-dependent manner.

## Introduction

Monocarboxylate transporter 1 (MCT1), encoded by *Slc16a1*, is a member of solute carrier family 16 (SLC16) and plays a crucial role in the transportation of lactate, pyruvate, ketone bodies, and short-chain fatty acids (SCFAs), as well as MCT1-targeted drugs in various tissues [1, 2]. MCT1 was first cloned as a mutant protein from Chinese hamster ovary cells in 1992 with an ability to transport mevalonate, a precursor for endogenous cholesterol synthesis [3]. In 1994, the wild-type form of MCT1 was identified to be a H^+^-coupled transporter for monocarboxylate, such as lactate and pyruvate [4]. MCT1 also has stereo-selectivity toward the isomers of lactate with a 10-fold preference for L-lactate over D-lactate [2, 5]. MCT1 is widely distributed in almost all human tissues [1]. In the heart and red skeletal muscles, high expression of MCT1 helps lactate to be used as oxidative fuel for mitochondrial respiration [6]. In the intestine, MCT1 is expressed both in the apical membrane and basolateral membrane of the intestinal epithelium [7]. In the kidney, MCT1 facilitates lactate influx in the proximal convoluted tubule [4]. MCT1 facilitates lactate efflux in many glycolytic cells, including white skeletal muscles, erythrocytes, astrocytes, oligodendrocytes, and immune cells, such as activated T-lymphocytes [8, 9]. MCT1 has been found to be ubiquitously overexpressed in multiple cancer cells and human tumor tissues that produce large amounts of lactate due to the Warburg effect [8].

In recent years, the functions of MCT1 in different tissues are beginning to be recognized, highlighting MCT1 as an important player in numerous physiological processes and diseases. Inactivation of MCT1 caused by mutations leads to defective utilization of ketone bodies and consequently results in recurrent ketoacidosis in children [10]. Recent studies have revealed that homozygous *Slc16a1* knockout in mice led to embryonic lethality, while the haploinsufficient mice (*Slc16a1*^+/−^) showed resistance to diet-induced obesity and associated metabolic perturbations [11]. Studies with heterozygous *Slc16a1*^+/−^ mice also indicated the role of MCT1 in the regulation of pH homeostasis and cellular energy homeostasis in skeletal muscles [12]. Due to the limitation of whole-body deletion of *Slc16a1,* tissue-specific *Slc16a1* knockout mouse models have been recently used to investigate the biological functions of MCT1 in various tissues/cells. Macrophage-specific deletion of *Slc16a1* can prevent M2-like polarization of macrophages, leading to impairment of muscle reperfusion and regeneration from ischemia [13]. *Slc16a1* deletion in macrophages was also found to affect peripheral nerve regeneration in mice [14]. *Slc16a1* deficiency in adipocytes stimulated macrophage-mediated inflammation, consequently leading to insulin resistance in peripheral tissues [15]. Hepatic deletion of *Slc16a1* aggravated high-fat diet (HFD)-induced obesity in female mice, but not in male mice [16].

As one of the largest organs in the body, the gut is the most important part of nutrient digestion and absorption. MCT1 in the intestinal epithelium has been proposed to be responsible for the absorption of SCFAs, which are produced by fermentation of dietary fiber by gut microbiota [17]. However, the role of intestinal MCT1 in regulating lactate transport and modulating glucose/lipid metabolism of the body is unclear. In this study, we generated a mouse model with specific deletion of *Slc16a1* in the intestinal epithelium and discovered that intestinal deficiency of MCT1 had metabolic phenotypes in a sex-dimorphic manner via modulation of lactate transport.

## Results

### Deficiency of MCT1 in intestinal epithelium improves glucose homeostasis in male mice

**Figure 1:**
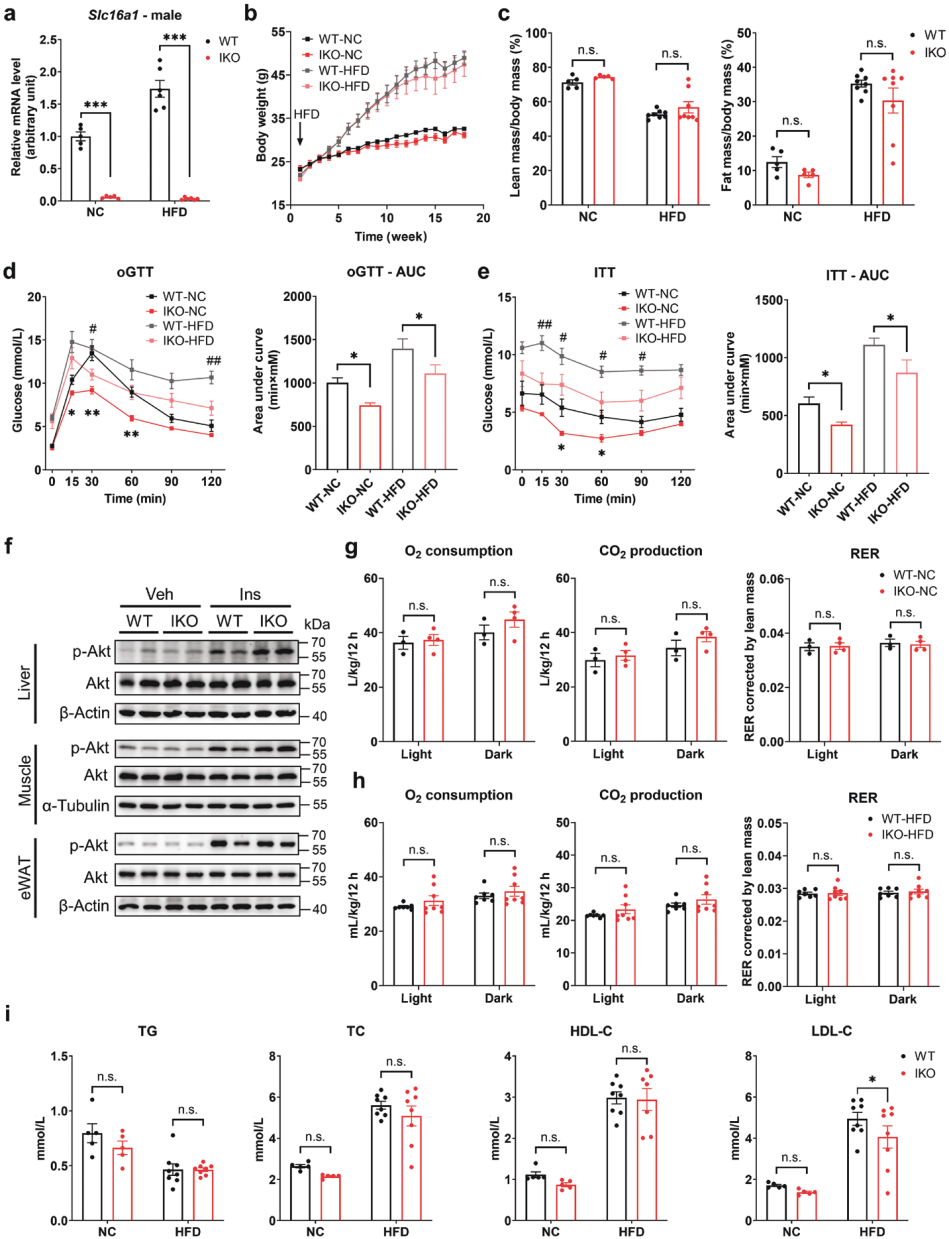
Deficiency of intestinal MCT1 improves glucose homeostasis in male mice. (a) *Slc16a1* mRNA level in WT and IKO male mice. *n* = 5 for the NC group, *n* = 6 for the HFD group. (b) Body weight curves of the mice fed with an NC diet or HFD. (c) Quantification of body fat mass ratio and lean mass ratio by MRI. (d and e) Oral glucose tolerance test (oGTT) and insulin tolerance test (ITT) of the mice with the corresponding area under the curve (AUC). ^*^ and ^#^ for comparison between WT and IKO groups under NC and HFD, respectively (^#^ for *P* < 0.05 and ^##^ for *P* < 0.01). (f) Western blotting of phosphorylated and total Akt levels in the liver, skeletal muscle, and epididymal white adipose tissues (eWAT). The mice were injected with or without 1 U/kg insulin for 15 min. (g) Results of metabolic cages to quantitate O_2_ consumption, CO_2_ production, and respiratory exchange ratio (RER) in the mice fed an NC diet. The quantifications were divided into light period (7:00 a.m. to 7:00 p.m.) and dark period (7:00 p.m. to 7:00 a.m.). *n* = 3 for WT group, *n* = 4 for IKO group. (h) Results of metabolic cages in mice fed HFD. (i) Plasma levels of TG, TC, HDL-C, and LDL-C. Data are shown as mean ± SEM. *n* = 5 for the NC groups, *n* = 8 for the HFD groups unless otherwise indicated. ^*^P < 0.05, ^**^P < 0.01, ^***^P < 0.001, n.s. for nonsignificant.

We generated a mouse model with specific deletion of *Slc16a1* in the intestinal epithelium (*Slc16a1*^IKO^ or IKO) by crossing villin-Cre mice with *Slc16a1*^fl/fl^ (Wild type, WT) mice [15]. The mice were fed with normal chow (NC) or a high-fat diet (HFD) to explore the potential functions of MCT1 on metabolic regulation in the presence or absence of diet-induced obesity. Knockout of *Slc16a1* in the intestinal epithelium was confirmed by quantitative real-time PCR to analyze the mRNA level of intestinal MCT1 in the *Slc16a1*^IKO^ mice (Fig. 1a). We also found that the mRNA levels of *Slc16a1* in different segments of the mouse intestine were significantly reduced in the *Slc16a1*^IKO^ mice (Supplementary Fig. S1).

In male mice, deletion of *Slc16a1* did not affect body weight, lean mass ratio, and fat mass ratio under both NC and HFD conditions (Fig. 1b and c). However, as compared to WT mice, the *Slc16a1*^IKO^ mice had significant improvement in glucose homeostasis as revealed by both the oral glucose tolerance test (oGTT) (Fig. 1d) and the insulin tolerance test (ITT) (Fig. 1e). Consistently, insulin sensitivity as measured by insulin-stimulated Akt phosphorylation was elevated by *Slc16a1* deletion in the liver, while not obviously changed in the skeletal muscle and epididymal fat (Fig. 1f). We also examined the energy metabolism of the mice using a metabolic cage and found that there were no changes in oxygen (O_2_) consumption, carbon dioxide (CO_2_) production, and respiratory exchange ratio (RER) under both NC and HFD conditions (Fig. 1g and h). *Slc16a1* deletion did not affect the plasma levels of triglyceride (TG), total cholesterol (TC), and high-density lipoprotein cholesterol (HDL-C), while reduced low-density lipoprotein cholesterol (LDL-C) only under HFD conditions (Fig. 1i). In conclusion, these data indicated that deletion of intestinal *Slc16a1* in male mice significantly improves glucose homeostasis but did not affect diet-induced obesity, metabolic rate, and most of the blood lipid parameters.

### Deletion of *Slc16a1* in intestinal epithelium aggravates HFD-induced obesity in female mice

**Figure 2:**
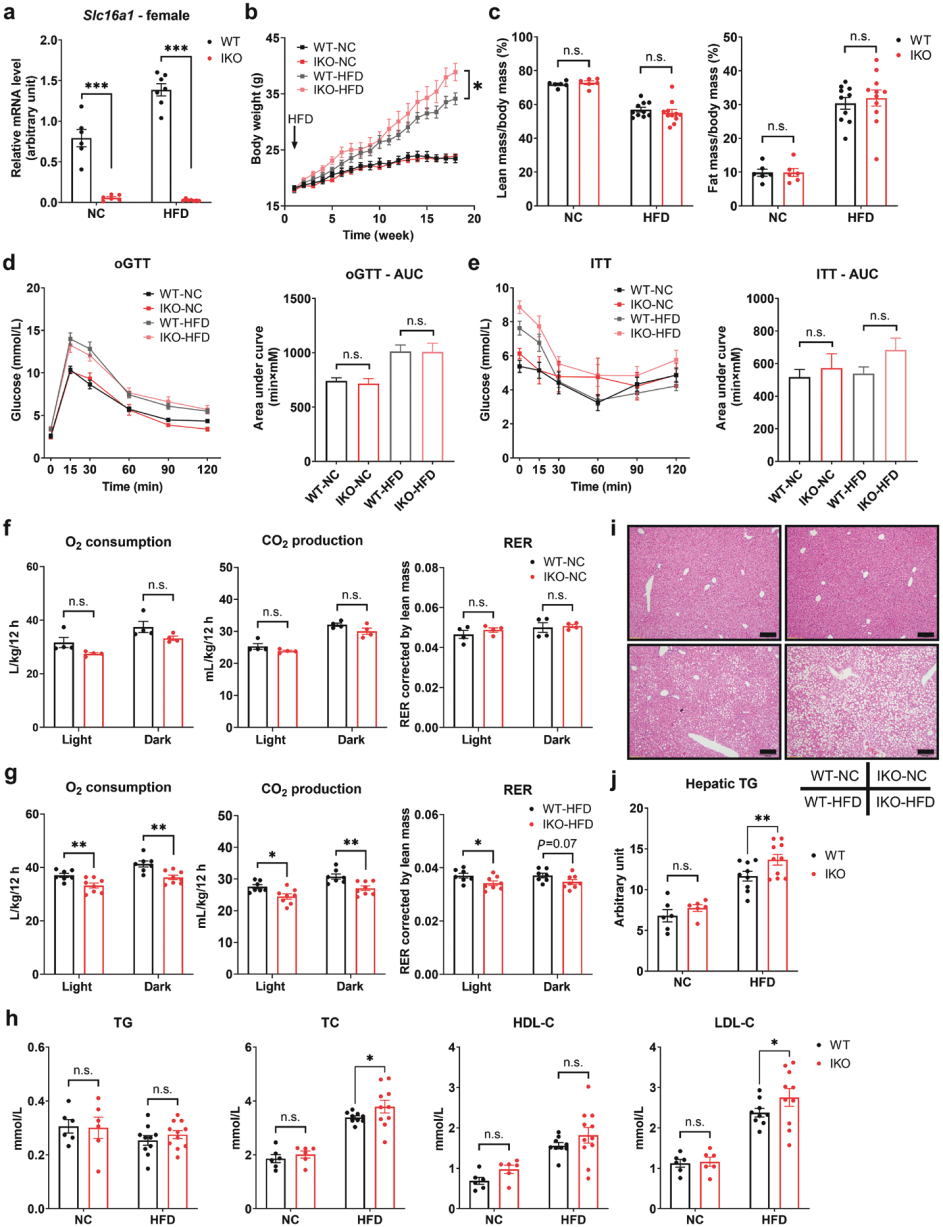
Deficiency of intestinal MCT1 accelerates body weight gain and impairs lipid profiles in HFD-fed female mice. (a) Results of RT-qPCR of *Slc16a1* mRNA levels in WT and IKO female mice. *n* = 6 for the NC group, *n* = 7 for the HFD group. (b) Body weight curves of WT and IKO female mice fed NC or HFD. (c) Quantification of body fat mass ratio and lean mass ratio by MRI scan. (d and e) Results of oGTT and ITT of the mice with corresponding AUC. (f) Analysis of metabolic cages to quantitate O_2_ consumption, CO_2_ production, and RER in female mice fed NC. *n* = 4 for each group. (g) Analysis of metabolic cages to quantitate O_2_ consumption, CO_2_ production, and RER in female mice fed HFD. *n* = 7 − 8 for each group. (h) Plasma levels of TG, TC, HDL-C, and LDL-C. (i) Representative HE staining of the liver in female mice. Scale bar, 50 μm. (j) Hepatic level of TG in female mice. Data are expressed as mean ± SEM. *n* = 6 for the NC groups, *n* = 9 − 11 for the HFD groups unless otherwise indicated. ^*^P < 0.05, ^**^P < 0.01, ^***^P < 0.001, n.s. for non significant.

We next examined the phenotype of the female mice with *Slc16a1* deletion in the intestinal epithelium (Fig. 2a). The female *Slc16a1*^IKO^ mice had an increased body weight gain as compared to WT mice under HFD condition (Fig. 2b), although without significant changes in lean mass ratio and fat mass ratio (Fig. 2c). In addition, glucose homeostasis as measured by oGTT and ITT was not altered by intestinal *Slc16a1* deletion in female mice (Fig. 2d and e). The metabolic rate of the female *Slc16a1*^IKO^ mice was significantly decreased only under HFD condition (Fig. 2f and g). The *Slc16a1*^IKO^ mice also had increases in blood TC and LDL-C levels under HFD condition (Fig. 2h). Hematoxylin-eosin (HE) staining of the liver revealed that HFD-induced lipid accumulation was elevated by *Slc16a1* deletion in female mice (Fig. 2i), together with increased TG level in the liver (Fig. 2j). Thus, the major phenotype of intestinal *Slc16a1-*deleted female mice was an aggravation of diet-induced obesity, associated with increases in blood cholesterol/LDL-C levels, a reduction in metabolic rate, and aggravated hepatic steatosis. On the other hand, intestinal *Slc16a1* deletion mainly led to the improvement of glucose homeostasis in male mice.

### RNA-sequencing (RNA-Seq) analysis of the intestine reveals sex-specific changes in gene expression affected by intestinal *Slc16a1* deletion

**Figure 3:**
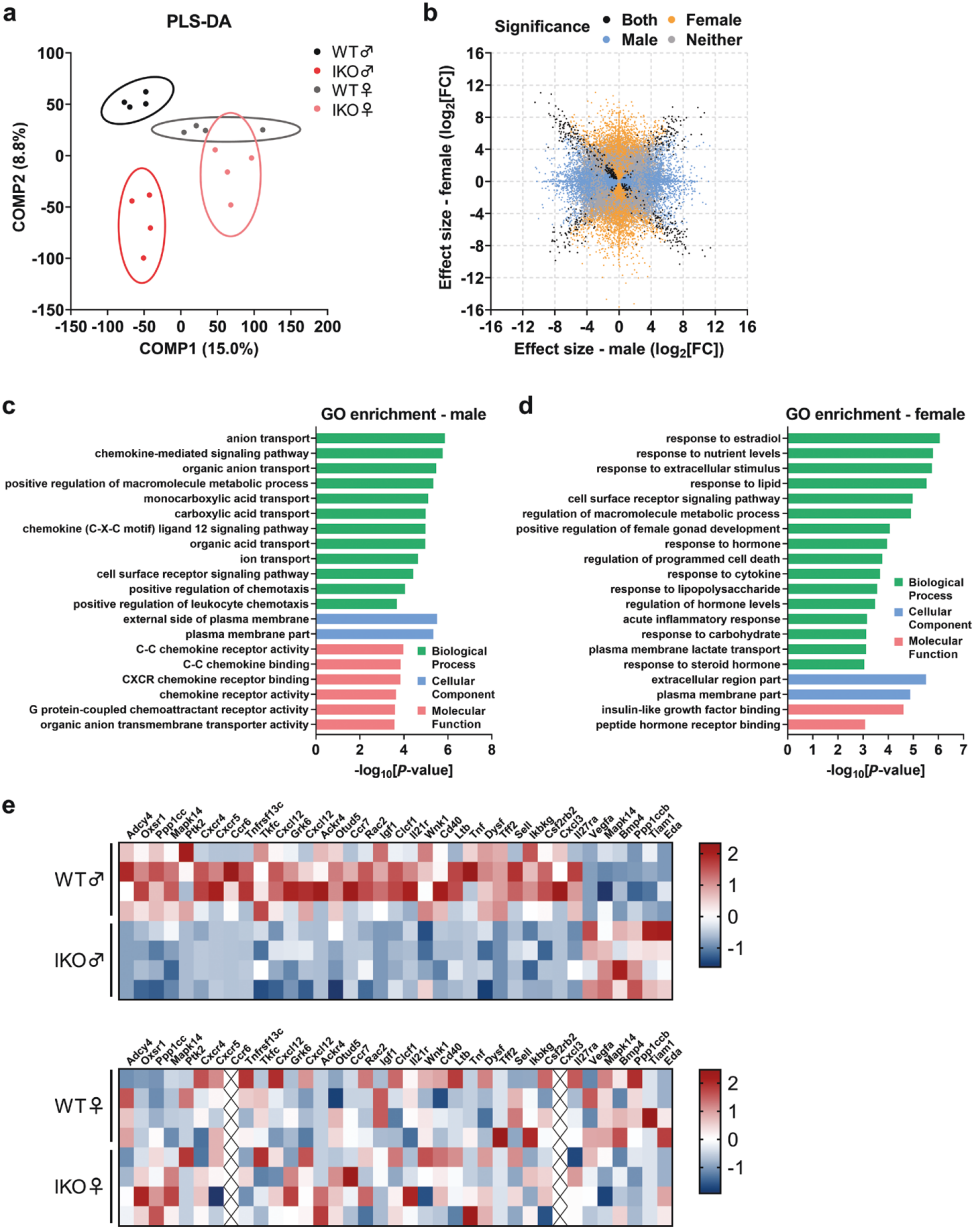
Transcriptome analysis reveals sex-discriminating features of the IKO mice. (a) Partial least squares discriminant analysis (PLS-DA) of all the sequencing samples. (b) Scatter plot showing effect size of intestinal *Slc16a1* depletion in male and female mice according to significance in one, both, or neither group. (c and d) GO enrichment analysis of the major DETs in male (c) and female (d) mice. (e) Heatmap analysis in male and female mice for transcripts that enriched in chemotaxis- and inflammation-associated GO terms of male mice.

To explore the potential mechanisms underlying the sex-specific metabolic phenotypes mediated by intestinal *Slc16a1* deletion, we applied RNA-Seq analysis with RNA isolated from the small intestines of WT and *Slc16a1*^IKO^ mice fed with HFD. The detailed pipeline for the analysis of the RNA-Seq data is illustrated in Supplementary Fig. S2. Partial least squares discriminant analysis (PLS-DA) revealed a clear separation between the transcriptomes of WT and *Slc16a1*^IKO^ samples, with the discrepancy being more evident in the male mice than the female mice (Fig. 3a). In total, 3800 transcripts in male mice and 3783 transcripts in female mice were significantly differentially expressed (|log_1.5_FC| ≥ 1 and *P*-value < 0.05) between WT and *Slc16a1*^IKO^ mice (Supplementary Fig. S3a and b). Although the numbers of differentially expressed transcripts (DETs) were similar in both sexes, there was little overlap among these DETs between the two sexes, as illustrated by a scatter plot of the effect size of intestinal *Slc16a1* knockout (Fig. 3b). Only 528 transcripts (~14% of total DETs) were significantly differentially expressed in both sexes, and 190 transcripts (~5% of total DETs) of which had the same trend (both upregulated or both downregulated). The other 338 transcripts (~9% of total DETs) had a completely divergent trend of expression. Thus, these results implied profound sex-specific gene expression profiles caused by intestinal *Slc16a1* deletion.

To identify the pathways enriched by intestinal *Slc16a1* knockout in each sex, we performed gene ontology (GO) and Kyoto encyclopedia of genes and genomes (KEGG) enrichment analysis based on the DETs. Before enrichment analysis, we screened out major DETs by cross-comparing the DETs with the GENCODE basic transcript set (release M25), which prioritizes full-length protein-coding transcripts (Supplementary Fig. S3c and d). After the elimination of certain outliers, we finally obtained 912 major DETs for male mice and 933 major DETs for female mice. In male mice, we found that intestinal *Slc16a1* knockout resulted in significantly altered GO terms related to ion/anion transport (Fig. 3c), which was in accordance with the function of MCT1. Moreover, we found that multiple enriched GO terms were closely related to chemokines or chemotaxis such as chemokine-mediated signaling pathway (*P* < 0.0001), C–C chemokine receptor activity (*P* = 0.0001), and C–C chemokine binding (*P* = 0.0001), and G-protein coupled chemoattractant receptor activity (*P* = 0.0003) (Fig. 3c). KEGG analysis also revealed that the most enriched pathways were centered in inflammatory cytokines or chemokines, including the cytokine–cytokine receptor interaction (*P* < 0.0001), inflammatory mediator regulation of transient receptor potential (TRP) channels (*P* = 0.0113), and chemokine signaling pathway (*P* = 0.0271) (Supplementary Fig. S3e). These results thus suggested that intestinal *Slc16a1* deletion mainly affected inflammation-related pathways in male mice.

However, intestinal *Slc16a1* disruption altered a discrepant set of GO terms or KEGG pathways in female mice (Fig. 3d and Supplementary Fig. S3f). The significantly enriched GO terms were associated with intestinal functions, including response to nutrient levels (*P* < 0.0001), response to lipid (*P* < 0.0001), and response to carbohydrate (*P* = 0.0007) (Fig. 3d). Similarly, multiple KEGG pathways were related to nutrient metabolism, such as protein digestion and absorption (*P* = 0.0092), vitamin digestion and absorption (*P* = 0.0216), riboflavin metabolism (*P* < 0.0001), and starch and sucrose metabolism (*P* = 0.0015) (Supplementary Fig. S3f). Intestinal *Slc16a1* deletion in female mice also resulted in significantly altered GO terms associated with responses to sex hormones, including response to estradiol (*P* < 0.0001), positive regulation of female gonad development (*P* < 0.0001), response to hormone (*P* = 0.0001), regulation of hormone levels (*P* = 0.0003), response to steroid hormone (*P* = 0.0009), and peptide hormone receptor binding (*P* = 0.0008) (Fig. 3d). Thus, the major changes of gene expression in intestinal *Slc16a1-*deleted female mice were pathways associated with nutrient response and sex hormones.

Next, we performed a detailed analysis with particular transcripts in the enriched pathways associated with inflammation and chemotaxis in male mice. A large proportion of these transcripts were downregulated in the male *Slc16a1*^IKO^ mice (Fig. 3e). However, most of these transcripts had similar expression patterns in both WT and female *Slc16a1*^IKO^ mice (Fig. 3e). Collectively, transcriptome analysis with the intestinal samples revealed a sex-specific difference in gene expression profiles, with inflammation-related genes being mainly altered in the male *Slc16a1*^IKO^ mice.

### Deficiency of intestinal MCT1 reduces local and systemic inflammation in male mice

**Figure 4:**
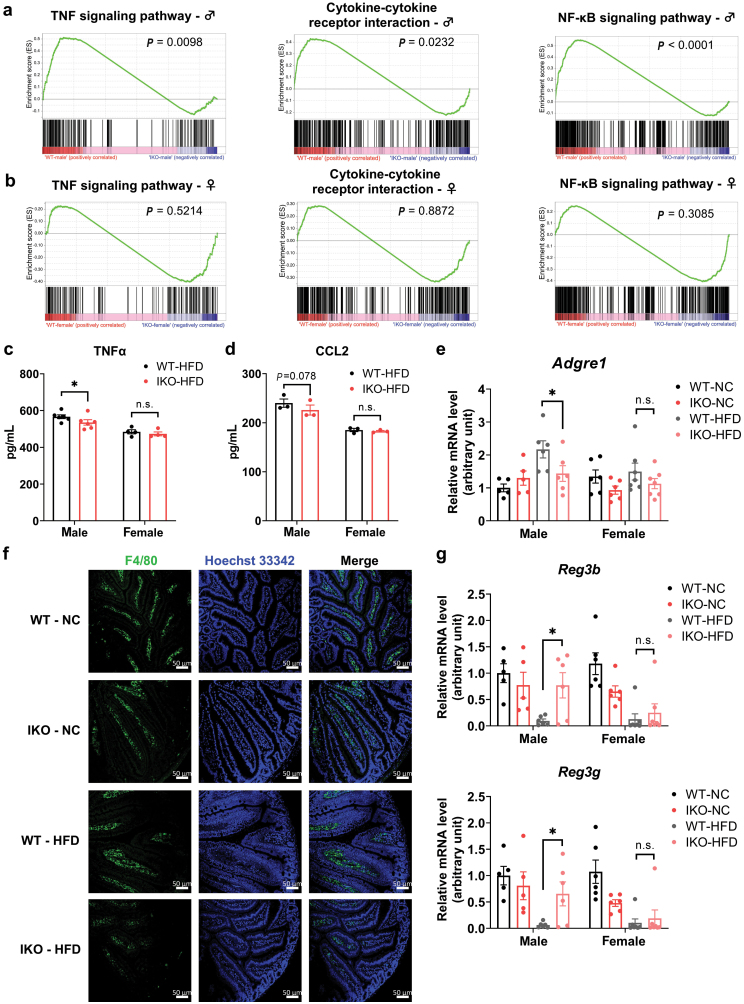
Deficiency of intestinal MCT1 reduces local and systemic inflammation in male mice. (a) Gene set enrichment analysis (GSEA) of the transcriptome data revealed significant enrichment in the pathways including TNF signaling pathway, cytokine-cytokine receptor interaction, and NF-κB signaling pathway in male mice. (b) Corresponding GSEA of the same pathways in female mice. (c and d) Plasma levels of TNFα (c) and CCL2 (d) determined by corresponding ELISA kits. *n* = 3 − 6 for each group. (e) Relative mRNA level of *Adgre1* by qPCR. *n* = 5 − 7 for each group. (f) Immunofluorescence staining of intestinal F4/80 in WT and IKO male mice. Scale bar, 50 μm. (g) mRNA levels of *Reg3b* and *Reg3g* detected by RT-qPCR. *n* = 5 − 7 for each group. Data are expressed as mean ± SEM. ^*^P < 0.05, n.s. for nonsignificant.

As implicated by the RNA-Seq analysis, inflammation-associated pathways were significantly reduced by intestinal *Slc16a1* deletion in male mice. To further explore this issue, we performed the gene set enrichment analysis (GSEA) based on all transcripts and discovered that several significantly enriched gene sets were related to inflammation. Deficiency of intestinal *Slc16a1* in male mice significantly downregulated the gene sets, including tumor necrosis factor (TNF) signaling pathway (*P* = 0.0098), cytokine-cytokine receptor interaction (*P* = 0.0232), and nuclear factor kappa B (NF-κB) signaling pathway (*P* < 0.0001) (Fig. 4a). However, these gene sets were not significantly enriched in female mice (Fig. 4b).

We also examined the blood levels of a few representative inflammatory cytokines. We observed a significant decrease in the level of circulating TNFα in the male *Slc16a1*^IKO^ mice fed HFD (Fig. 4c). The blood level of C–C motif chemokine ligand 2 (CCL2) also had a tendency of reduction in HFD-fed male *Slc16a1*^IKO^ mice (Fig. 4d). We analyzed the transcriptome data with sex-discriminating genes and noted that the macrophage marker *Adgre1* (F4/80) had a significant decrease in HFD-fed male *Slc16a1*^IKO^ mice, but not in female mice (Fig. 4e). Consistently, the immunofluorescence assay revealed a clear reduction in the number of intestinal macrophages that were positive for F4/80 antibody in male *Slc16a1*^IKO^ mice as compared to WT mice (Fig. 4f). In addition, we also found that the expression of two members of the Reg3 family, *Reg3b* and *Reg3g*, had sex-specific changes in mRNA level (Fig. 4g). *Reg3b* and *Reg3g* encode proteins Reg3β and Reg3γ that belong to C-type lectins and function as antimicrobial peptides (AMPs) which play an important role in anti-inflammatory immune responses [18]. We observed that HFD significantly decreased the mRNA levels of *Reg3b* and *Reg3g* in both sexes. However, intestinal *Slc16a1* knockout significantly increased *Reg3b* and *Reg3g* levels in HFD-fed male mice but not in female mice (Fig. 4g). Collectively, these results reveal that deficiency of intestinal MCT1 in male mice alleviates intestinal inflammatory response and systemic inflammation, likely contributing to the improvement of glucose metabolism in these mice.

### MCT1 affects extracellular lactate levels and the inflammatory response of macrophages

**Figure 5:**
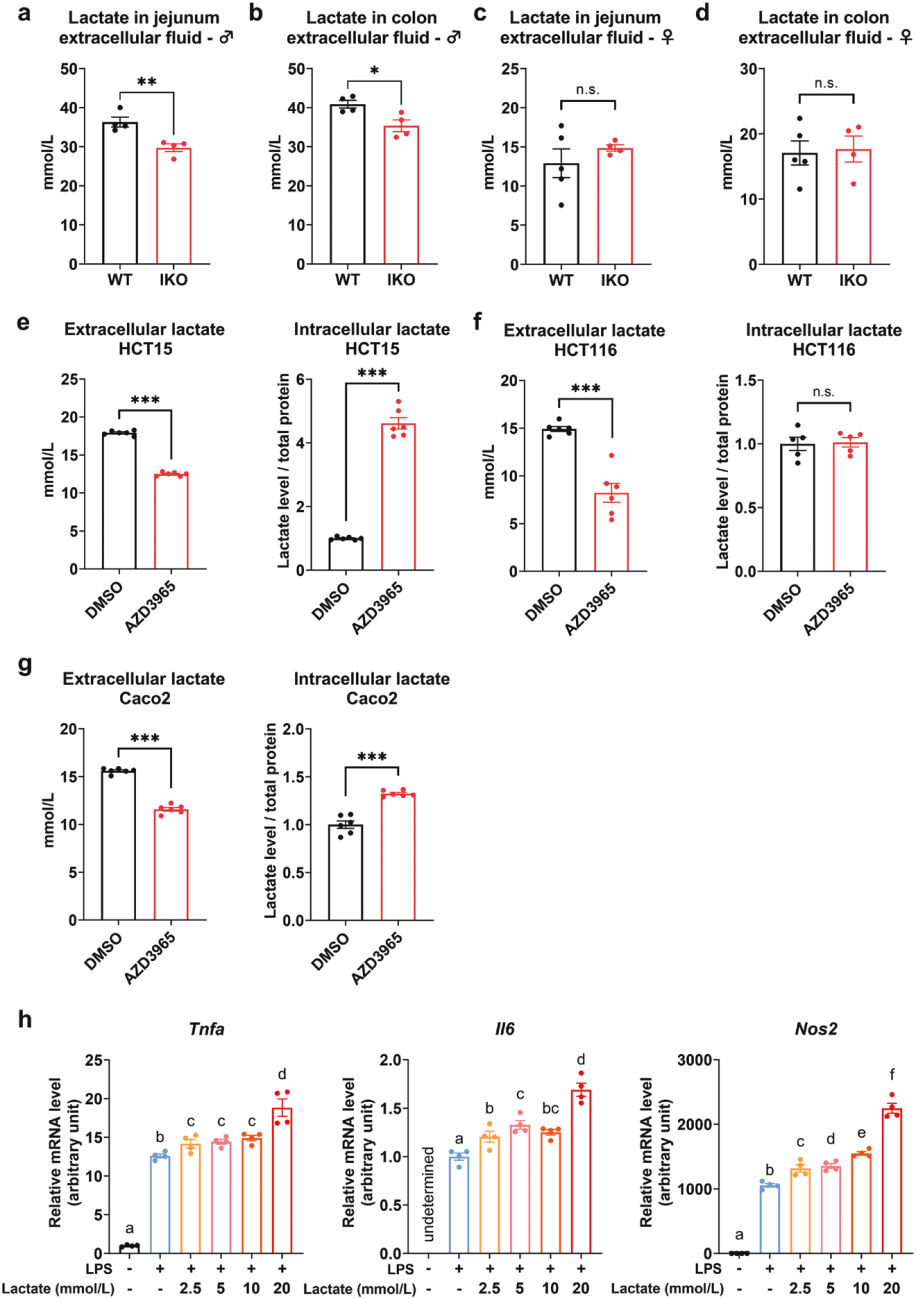
MCT1 is involved in the efflux of lactate which modulates macrophage activity. (a and b) Determination of lactate levels in the extracellular fluid of jejunum (a) and colon (b) in male mice. *n* = 4 for each group. (c and d) Determination of lactate levels in the extracellular fluid of jejunum (c) and colon (d) in female mice. *n* = 5 for WT group, *n* = 4 for the IKO group. (e–g) Determination of extracellular and intracellular lactate levels in HCT15 cells (e), HCT116 cells (f), and Caco2 cells (g) upon treatment with MCT1 inhibitor AZD3965. *n* = 5 − 6 for each group. (h) mRNA levels of *Tnfa*, *Il6*, and *Nos2* in macrophage of RAW264.7 cells treated with LPS and different doses of sodium L-lactate. *n* = 4 for each group. Data are expressed as mean ± SEM. ^*^P < 0.05. ^**^P < 0.01. ^***^P < 0.001, n.s. for nonsignificant.

A recent study indicated that adipocyte-derived lactate is a signaling metabolite that potentiates macrophage inflammation [19]. We investigated whether lactate derived from intestinal epithelial cells affects the functions of macrophages. First, we extracted the interstitial fluid from the jejunum and colon of WT and *Slc16a1*^IKO^ mice in both sexes. The lactate level in extracellular fluid was significantly decreased in the intestines of *Slc16a1*^IKO^ male mice (Fig. 5a and b), indicating that lactate efflux into the intestinal microenvironment was mediated by MCT1 in male mice. However, the lactate concentration in the extracellular fluid was not altered by *Slc16a1* deletion in the female mice (Fig. 5c and d).

We next analyzed intestinal cell lines HCT15, HCT116, and Caco2 that originated from human intestinal epithelium. The cells were treated with AZD3965, a well-characterized inhibitor of MCT1 [20]. Pharmacological inhibition of MCT1 significantly decreased the extracellular lactate level, while increasing the intracellular lactate level in these cells (Fig. 5e–g), further indicating that MCT1 was mainly responsible for lactate efflux in the intestinal epithelial cells. To evaluate the potential function of lactate on the macrophage, we treated RAW264.7 macrophage with lipopolysaccharides (LPS) and different concentrations of lactate. We found that lactate could potentiate LPS-induced expression of a few key marker genes involved in pro-inflammatory responses including *Tnfa*, *Il6,* and *Nos2* in a dose-dependent manner (Fig. 5h). Collectively, these results suggest that the local lactate level in the intestinal microenvironment is modulated by MCT1 in the intestinal epithelium. Furthermore, lactate in such microenvironment plays an important role in modulating inflammatory responses of pro-inflammatory macrophages.

### Estrogen abolishes the difference in glucose homeostasis between *Slc16a1* ^IKO^ and WT male mice while lowers interstitial lactate level in intestine

**Figure 6:**
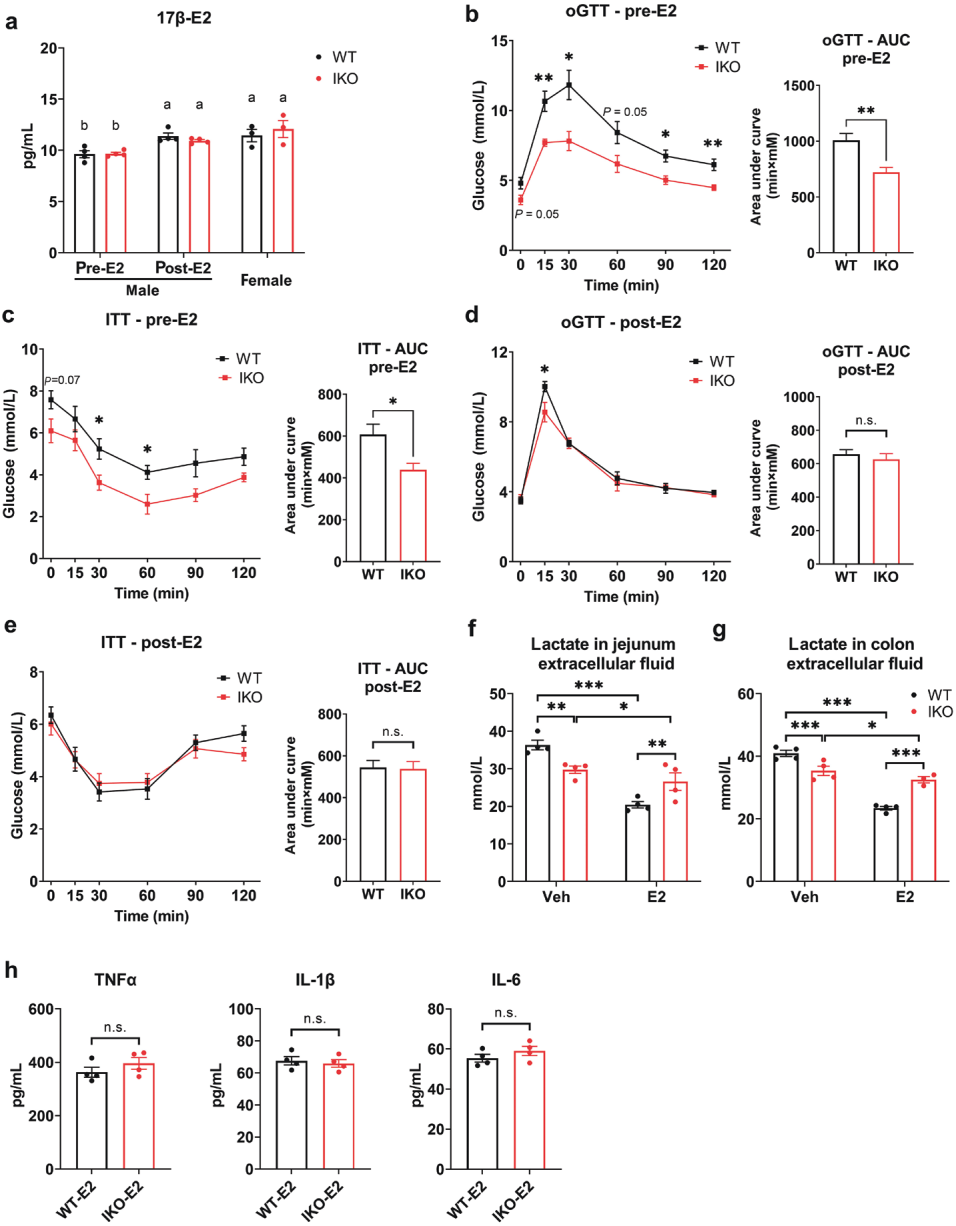
Estrogen treatment abolishes the difference in glucose homeostasis between IKO and WT male mice. (a) Circulating E2 levels before and after E2 administration. *n* = 3 − 4 for each group. (b) oGTT of male mice with corresponding AUC before E2 treatment. *n* = 4 − 7 for each group. (c) ITT of male mice with corresponding AUC before E2 treatment. *n* = 4 − 7 for each group. (d) oGTT of male mice with corresponding AUC after injection of E2. (e) ITT of male mice with corresponding AUC after injection of E2. (f and g) Determination of lactate levels in the extracellular fluid of jejunum and colon in male mice with or without E2 treatment. *n* = 4 for each group. (h) Circulating levels of TNFα, IL-1β, and IL-6 in E2-treated male mice determined by ELISA. *n* = 4 for each group. Data are expressed as mean ± SEM. ^*^P < 0.05. ^**^P < 0.01, n.s. for nonsignificant.

Estrogens are found to have a fundamental role in host physiology especially in the control of energy homeostasis and glucose metabolism [21]. Estrogen in females is considered to have a protective role in alleviating insulin resistance and inflammatory responses [22–24]. As we observed that glucose homeostasis was only improved in *Slc16a1*^*IKO*^ male mice but not in *Slc16a1*^*IKO*^ female mice, we hypothesized that estrogen may play a role in such a sex-dimorphic effect. To explore this hypothesis, we administrated 17β-estradiol (E2) to the male mice through intraperitoneal (i.p.) injection. Firstly, we examined the circulating E2 levels by enzyme-linked immunosorbent assay (ELISA). As expected, E2 was significantly elevated in both WT and *Slc16a1*^IKO^ male mice after E2 injection (Fig. 6a). E2 level was significantly higher in the female mice than the male mice, while not different between WT and *Slc16a1*^IKO^ mice (Fig. 6a). We analyzed the glucose metabolism of the mice via measurement of oGTT and ITT. As expected, *Slc16a1*^IKO^ male mice had improvement in both glucose tolerance and insulin sensitivity without E2 treatment (Fig. 6b and c). However, E2 treatment could completely abolish the difference in glucose homeostasis between *Slc16a1*^IKO^ and WT male mice (Fig. 6d and e). We next examined the lactate concentration in intestinal extracellular fluid from these mice. E2 treatment significantly decreased extracellular lactate levels in the intestinal interstitial fluid of male mice (Fig. 6f and g). In addition, the levels of inflammatory cytokines including TNFα, interleukin (IL)-1β, and IL-6 were not different between WT and *Slc16a1*^IKO^ mice after E2 treatment (Fig. 6h). These results thus indicate that estrogen has a critical role in mediating the modulatory role of intestinal *Slc16a1* deletion on glucose homeostasis in male mice.

### Deficiency of intestinal MCT1 blocks the transport of lactate and SCFAs from the intestine to the portal vein

**Figure 7:**
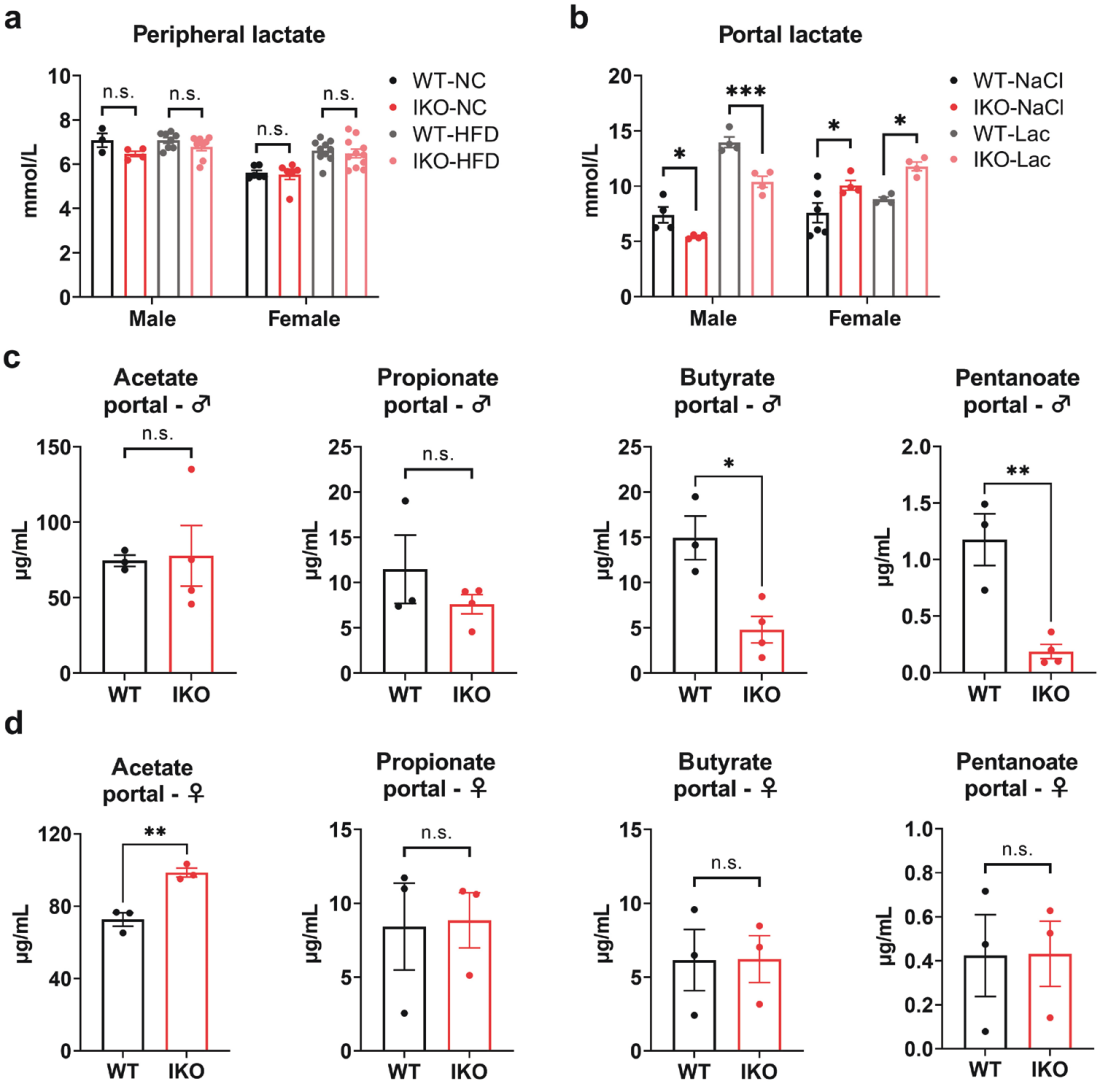
Deficiency of intestinal MCT1 blocks the transport of lactate and SCFAs from the intestine to the portal vein. (a) Determination of plasma lactate levels in WT and IKO mice. *n* = 3–4 for male mice fed NC, *n* = 8 for male mice fed HFD, *n* = 6 for female mice fed NC, *n* = 10–11 for female mice fed HFD. (b) Determination of lactate level in portal vein blood after oral administration of sodium L-lactate in WT and *Slc16a1*^IKO^ mice. *n* = 4–6 for each group. (c) Detection of acetate, propionate, butyrate, and pentanoate in portal vein blood from male mice treated with inulin. *n* = 3–4 for each group. (d) Detection of acetate, propionate, butyrate, and pentanoate in portal vein blood from female mice treated with inulin. *n* = 3 for each group. Data are expressed as mean ± SEM. ^*^P < 0.05. ^**^P < 0.01. ^***^P < 0.001, n.s. for nonsignificant.

As previous studies have indicated that MCT1 is involved in the transport of lactate and SCFAs in the intestine, we next investigated whether disruption of MCT1 in the intestine affected the transport of these metabolites. We first detected the blood lactate levels in WT and *Slc16a1*^IKO^ mice. Disruption of intestinal MCT1 did not alter the circulating lactate level (Fig. 7a). We next analyzed the lactate level in the portal vein that collects blood from the gastrointestinal tract. The mice were treated by oral gavage with a single dose of sodium L-lactate or saline. The lactate level in the portal vein blood was reduced in *Slc16a1*^IKO^ male mice under both NC and HFD conditions (Fig. 7b). In contrast, the lactate level in the portal vein blood was increased in *Slc16a1*^IKO^ female mice compared with WT littermates (Fig. 7b). These results thus indicate a sharp difference in MCT1-mediated lactate transport in the intestine between male and female mice.

We next examined the SCFA levels in the portal vein blood in the mice with oral gavage with inulin solution. Inulin is a type of dietary fiber that is fermented by specific gut bacteria to produce SCFAs. *Slc16a1*^IKO^ male mice were found to have marked reductions of butyrate (*P* < 0.05) and pentanoate (*P* < 0.01) in the portal vein blood (Fig. 7c), but not for acetate and propionate (Fig. 7c). However, *Slc16a1*^IKO^ female mice did not show similar reductions in these SCFAs (Fig. 7d). The level of acetate even had a significant increase in *Slc16a1*^IKO^ female mice (Fig. 7d). Taken together, these results indicate intestinal MCT1 depletion blocks lactate and SCFA transport from the intestine to the portal vein only in *Slc16a1*^IKO^ male mice.

### Intestinal *Slc16a1* deletion improves glucose homeostasis partly through modulation of gut microbiota in male mice

**Figure 8:**
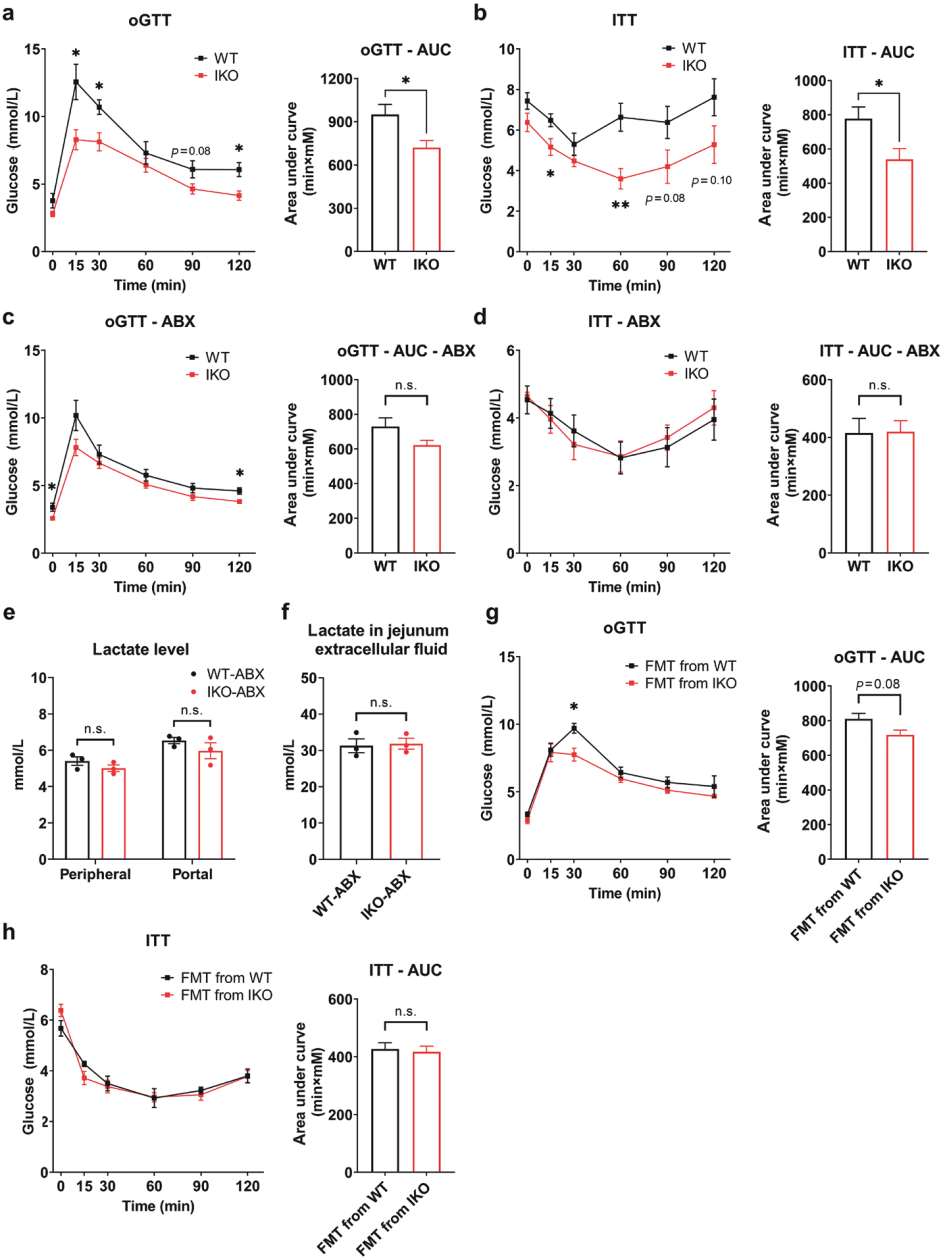
Deficiency of intestinal MCT1 improves glucose homeostasis through modulation of gut microbiota in male mice. (a) oGTT of male mice with corresponding AUC before antibiotic treatment. (b) ITT of male mice with corresponding AUC before antibiotic treatment. (c) oGTT of male mice with corresponding AUC after antibiotic-induced microbiota depletion. (d) ITT of male mice with corresponding AUC after antibiotic treatment. (e) Determination of peripheral and portal lactate levels in antibiotic-treated male mice. *n* = 3 for each group. (f) Determination of lactate level in the extracellular fluid of jejunum in antibiotic-treated male mice. *n* = 3 for each group. (g) oGTT of mice received FMT from WT and IKO male mice with corresponding AUC. *n* = 4–6 for each group. (h) ITT of mice received FMT from WT and IKO male mice with corresponding AUC. *n* = 4–6 for each group. Data are expressed as mean ± SEM. ^*^P < 0.05. ^**^P < 0.01, n.s. for non-significant.

As gut microbiota plays a key role in modulating intestinal inflammation and we observed that the local inflammation and glucose homeostasis were improved by MCT1 in male mice, we hypothesized that gut microbiota had a functional role underlying these phenotypes. We applied antibiotic treatment to eliminate the gut microbiota in WT and *Slc16a1*^IKO^ male mice fed an NC diet, and examined glucose metabolism by oGTT and ITT. Before antibiotic treatment, as expected, we found that deficiency of intestinal MCT1 significantly improved glucose tolerance and insulin sensitivity (Fig. 8a and b). After the elimination of the gut microbiota, depletion of intestinal *Slc16a1* no longer improved glucose tolerance and insulin sensitivity (Fig. 8c and d). Meanwhile, the deletion of intestinal *Slc16a1* no longer affected the lactate level both in portal blood and in intestinal interstitial fluid after antibiotic treatment in the male mice (Fig. 8e and f). These observations thus suggest that gut microbiota might have an impact on lactate efflux from the gut epithelium to the interstitial space and portal vein. However, further evaluation of flow cytometry using isolated intestinal lamina propria lymphocytes (LPLs) showed that the number of F4/80^+^CD11b^+^ macrophages (gated on CD45^+^MHCII^+^ cells) was still reduced in *Slc16a1*^IKO^ male mice compared to WT mice after antibiotic treatment (Supplementary Fig. S4a and b). Reduction of macrophage in *Slc16a1*^IKO^ mice was also found by immunofluorescence staining of F4/80 in the colon (Supplementary Fig. S4c). Also, the expression of IL-1β and IL-6 was reduced by intestinal *Slc16a1* deletion regardless of antibiotic treatment (Supplementary Fig. S4d). These results thus indicate that antibiotic treatment cannot completely abrogate intestinal *Slc16a1* deletion-mediated reduction of inflammation in male mice.

Furthermore, we performed fecal microbiota transplantation (FMT) to determine whether the phenotype of improved glucose homeostasis in *Slc16a1*^IKO^ male mice could be transferred to other mice. Compared with the mice that received FMT from WT male mice, the mice that received FMT from *Slc16a1*^IKO^ male mice had a slight improvement in glucose tolerance (Fig. 8g), but not insulin sensitivity (Fig. 8h). Collectively, these data suggest that gut microbiota are only partially involved in MCT1-mediated regulation of glucose homeostasis in male mice.

**Figure 9:**
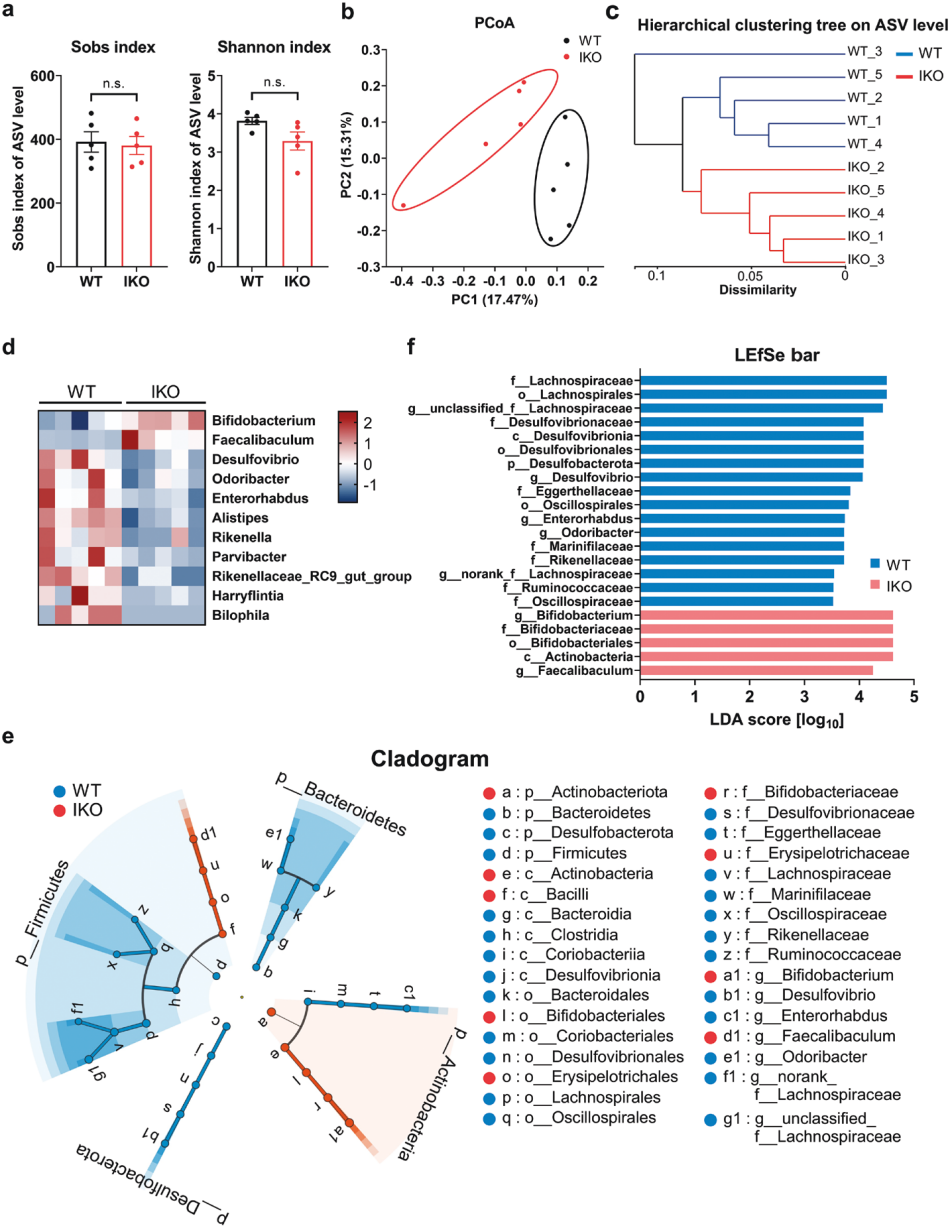
The impact of intestinal MCT1 deletion on the gut microbiota of male mice. (a) Sobs index and Shannon index of gut microbiota in male mice. (b) Principal coordinates analysis (PCoA) of the microbiota samples from male mice. (c) Hierarchical clustering of gut microbiota on amplicon sequence variant (ASV) level in male mice. (d) Community heatmap at genus level showing different bacteria between WT and IKO male mice with adjusted *P* < 0.05. (e and f) Linear discriminant analysis (LDA) effect size (LEfSe) analysis (e) and corresponding LDA scores (f) in WT and IKO mice. p, phylum; c, class; o, order; f, family; g, genus. Data are expressed as mean ± SEM. *n* = 5 for each group. n.s. for nonsignificant.

To illustrate whether or not gut microbiota were altered by intestinal *Slc16a1* deletion, we performed 16S rRNA gene sequencing with the feces collected from WT and *Slc16a1*^IKO^ male mice fed an NC diet. The richness and diversity of microbial community shown by the Sobs index and Shannon index respectively in the two groups had no apparent changes (Fig. 9a). Principal co-ordinates analysis (PCoA) by unweighted UniFrac distance was performed to assess the β diversity of the gut microbiota. Notably, the gut microbiota exhibited significant structural modulation upon intestinal *Slc16a1* deletion (Fig. 9b). Hierarchical clustering analysis based on β diversity distance matrix also illustrated a structural rearrangement of gut microbiota between the two groups (Fig. 9c). As for microbial composition, at the genus level, the proportion of *Bifidobacterium* and *Faecalibaculum* were significantly increased in *Slc16a1*^IKO^ mice compared to WT mice (*P* < 0.05, Fig. 9d). We also found that several bacterial genera were declined in the *Slc16a1*^IKO^ mice (Fig. 9d) such as *Desulfovibrio* (*P* < 0.05) and *Odoribacter* (*P* < 0.05) which were often considered to be opportunistic pathogens. We then performed linear discriminant analysis (LDA) effect size (LEfSe) analysis and identified a few microbial features which significantly contributed to the overall differences (Fig. 9e and f). Consistently, intestinal *Slc16a1* deficiency was associated with the reduction of bacteria from genera *Desulfovibrio*, *Enterorhabdus,* and *Odoribacter*, along with increases of bacteria from genera *Bifidobacterium* and *Faecalibaculum* (Fig. 9e and f). In addition, microbes from *Lachnospiraceae*, *Ruminococcaceae*, and *Oscillospiraceae* also had significant reductions in *Slc16a1*^IKO^ mice (Fig. 9e and f). These data thus suggest that intestinal *Slc16a1* deletion is associated with certain changes in gut microbiota in male mice.

## Discussion

Our studies demonstrate that lactate serves as a critical link that associates intestinal inflammation with systemic glucose homeostasis. Previous studies have indicated that the substrates of MCT1 include multiple endogenous metabolites, such as lactate, pyruvate, SCFAs, and β-hydroxybutyrate [2]. These compounds, especially lactate, can serve as fuel sources to feed the tricarboxylic acid (TCA) cycle for ATP production [25]. Thus, it is not surprising that MCT1 could play a role in energy metabolism *in vivo*. As the most important organ for nutrient digestion and absorption, the intestine is considered to be the largest immune organ as well as endocrine organ. Our study indicated that lactate transport in the intestinal epithelium is mainly involved in the modulation of local inflammation in male mice. In particular, interstitial lactate transported through MCT1 regulates the activities of intestinal macrophages and such change of macrophages contributes to systemic inflammation, consequently affecting glucose tolerance and insulin sensitivity in peripheral tissues in male mice.

It is well-accepted that inflammation is closely related to the development of metabolic diseases and insulin resistance. Our study found that *Slc16a1*^IKO^ male mice had improved glucose homeostasis with reduced levels of local and systemic inflammation. Specifically, intestinal deficiency of MCT1 in male mice was linked with decreased local lactate level in intestinal interstitial space, reduced microphage infiltration, reduced production of inflammatory cytokines, and elevated expression of antimicrobial peptides in male mice. Our *in vitro* experiment indicated that elevated lactate levels had a positive role in LPS-stimulated production of pro-inflammatory cytokines from macrophages (Fig. 5h). The circulating levels of pro-inflammatory cytokines TNFα and CCL2 were reduced in *Slc16a1*^IKO^ male mice under HFD condition (Fig. 4c and d). In addition, the circulating levels of IL-1β and IL-6 were reduced in male mice under NC conditions (Supplementary Fig. S4d). We propose that the reduced local and systemic inflammation by intestinal *Slc16a1* deletion in male mice is contributed by reduced lactate concentration in the interstitial microenvironment due to MCT1 deficiency. Considering the unique function of MCT1 in modulating intestinal inflammation, our results highlight that intestinal MCT1 could serve as a potential drug target for metabolic disorders.

It is noteworthy that the expression of antimicrobial peptides of the Reg3 family in the intestine was augmented by intestinal *Slc16a1* deletion in the male mice under HFD conditions (Fig. 4g). It has been previously reported that Reg3 family members can protect against diabetes and alcoholic steatohepatitis [26, 27]. *Reg3b*^−/−^ and *Reg3g*^−/−^ mice had increased F4/80 positive cells, upregulated gene expression of chemokines C-X-C motif ligand 1 (*Cxcl1*), *Ccl2*, and *Cxcl5*, and elevated TNFα protein levels in the liver following ethanol feeding [26]. *Reg3b*-deficient mice also display elevated inflammation in a dextran sulfate sodium (DSS)-induced colitis model [28]. It is thus possible that the improvement of glucose homeostasis in the intestinal *Slc16a1*-deleted male mice is partly mediated by the upregulation of the Reg3 family members. This is an issue worthy of investigation in the future.

Sexual dimorphism is a common phenomenon in most animals. This sex asymmetry has been attributed to the differential effects of sex hormones and genetic differences, which have substantial impacts on systemic metabolism and metabolic diseases [29]. Sexual dimorphism has been found to exist in many organs/tissues such as adipose tissues, liver, skeletal muscle, and intestine [30–33]. A recent study has indicated that intestinal lipid absorption and lymphatic transport differ between the sexes [33]. Ovarian hormone reduces dietary lipids absorption and lymphatic transport in the intestine, contributing to the reduced risk of atherosclerosis in females [33]. In most cases, males are generally more susceptible to impaired glucose metabolism with lower insulin sensitivity than females [34, 35]. In this study, we have discovered that glucose metabolism is only improved in male mice with MCT1 deficiency in the intestine. Furthermore, we found that estrogen administration could abolish the difference in glucose homeostasis between *Slc16a1*^IKO^ and WT male mice, while reducing interstitial lactate levels in the intestine, suggesting estrogen as a key factor for the observed sexual dimorphism in our study. We also speculate that intestinal MCT1 depletion could not result in an obvious effect on glucose metabolism in female mice owing to the protective effect of E2. In other words, the effect of intestinal MCT1 on glucose homeostasis is only permissive in male mice in which the estrogen level is relatively low.

Our study also demonstrates that intestinal MCT1 is involved in the transport of SCFAs in male mice. SCFAs are derived from microbial fermentation of dietary fibers and have profound impacts on metabolic health [36]. Our study suggests that disruption of intestinal MCT1 blocks SCFA absorption in a sex-dimorphic manner. This raises the question of whether SCFA absorption is different in different sexes. Furthermore, the physiologic impact of SCFAs on the body is likely dependent on the sex. It is known that different SCFAs have complicated effects on the health of the host [36]. For example, SCFAs are commonly associated with metabolic benefits and improvement in immune regulation. However, as important energy sources, excessive SCFAs are associated with increased energy input in the intestine, which could contribute to obesity [37]. Among the SCFAs, butyrate has been extensively investigated as an anti-inflammatory agent at least partly by inhibiting histone deacetylases (HDACs) [38]. However, several studies have shown that high levels of SCFAs can cause dysregulated T-cell responses and promote inflammatory responses in mice [39, 40]. Under these considerations, downregulation of the local and systemic inflammation in *Slc16a1*^IKO^ male mice might result from a decrease in intestinal absorption of SCFAs. It is thus important to elucidate in the future whether a particular SCFA can modulate the functions of intestinal macrophages and local inflammation.

G protein-coupled receptor 81 (GPR81) was found to mediate gut microbe-derived lactate signals and affect intestinal stem cell proliferation in a previous study [41]. However, we did not observe significant changes in villi length, crypt height, and other architecture of the intestine between WT and *Slc16a1*^IKO^ mice in both sexes (data not shown). Furthermore, we performed immunofluorescence staining of Ki67, a marker of cell proliferation, in the intestinal sections of the mice. We found that Ki67 staining in the base of the crypts was not altered by intestinal *Slc16a1* deletion (data not shown). In addition, the lactate level in the content of small intestines and GPR81 mRNA level were not changed in *Slc16a1*^IKO^ mice (data not shown), suggesting that the proliferation of intestinal stem cells is not likely affected by intestinal *Slc16a1* deletion.

The gut microbiota is an enormous ecosystem and has a marked impact on the host immune system and metabolic health [42, 43]. Gut microbiota dysbiosis is often associated with dysregulated glucose metabolism and metabolic syndrome. It was recently discovered that the sex hormone androgen could deteriorate glucose homeostasis by modulating gut microbiota, thus contributing to sexual dimorphism in glucose metabolism [34]. In our study, we found that the sex-specific effects on glucose metabolism in the *Slc16a1*^IKO^ male mice were nullified when gut microbiota was depleted by an antibiotic cocktail. Furthermore, FMT from the *Slc16a1*^IKO^ male mice partially recapitulated the improvement of glucose tolerance. These results indicate that gut microbiota is an important sex-discriminating factor for host glucose homeostasis upon intestinal depletion of *Slc16a1*. Indeed, there was a substantial alteration in the composition of the microbial community in the intestinal *Slc16a1-*deleted mice. Disruption of MCT1 elevated the proportions of *Bifidobacterium* and *Faecalibaculum* in gut microbiota. Numerous animal studies and human studies have suggested that supplementation of *Bifidobacterium* strains could improve glucose metabolism, mitigate inflammation, and reverse HFD-induced metabolic disorders [44]. *Faecalibaculum*, featured by *F. rodentium*, was also found to produce lactic acid as a major metabolic end product [45]. Lactic acid-producing bacteria are often considered to promote metabolic health [46]. Meanwhile, certain potentially harmful bacteria such as *Desulfovibrio* and *Odoribacter* declined after intestinal *Slc16a1* disruption. *Desulfovibrio* belongs to the sulfate-reducing bacteria (SRB) which are a group of anaerobic microbes metabolizing sulfate into hydrogen sulfide (H_2_S) [47]. Recent studies have indicated that the SRB are potential endotoxin producers leading to a low-grade chronic inflammation, causing obesity and diabetes [48]. In an animal study, *Odoribacter* was identified exclusively in the obese and diabetic *db/db* mice as compared to lean control mice [49]. Taking these discoveries into consideration, we therefore propose that intestinal disruption of *Slc16a1* might bring about beneficial effects to glucose homeostasis partly by reshaping the structure of gut microbiota.

In conclusion, our study uncovers the essential role of intestinal MCT1 in regulating intestinal inflammation and metabolic profiles in a sex-dimorphic manner. In the intestinal MCT1-deficient mice, we discovered sex-dependent alterations in glucose tolerance/insulin sensitivity, diet-induced obesity, transport of monocarboxylate including lactate and SCFAs, local and systemic inflammation, and gut microbiota. We also provided preliminary evidence indicating that estrogen might partly underlie the sex dimorphism in the mice. Our findings further corroborate the concept that metabolic homeostasis is differently regulated in the two sexes. This work also strengthens the demand for developing sex-specific medicines for metabolic disorders and highlights the importance of stratifying patients based on sex in the management and treatment of metabolic diseases.

## Materials and methods

The detailed materials and methods are described in Supplementary Materials.

### Mice

All animal experimental protocols were approved by Institutional Animal Care and Use Committee Institutional Animal Care and Use Committee of the Shanghai Institute of Nutrition and Health, Chinese Academy of Sciences (CAS) with an approval number SINH-2020-CY-1.

### Analysis of SCFAs

Mice were pre-treated with inulin (Titan, China) solution (5 g/kg/day) for seven consecutive days by oral gavage. Determination of SCFAs including acetic, propionic, butyric, and pentanoic acids was performed by gas chromatography.

### Estrogen administration

17β-Estradiol (E2) (MedChemExpress, USA) was dissolved in olive oil. The mice received an i.p. injection of E2 (10 mg/kg body weight) every other day for 2 weeks.

### Lactate measurement

Lactate from samples of plasma, cell media, cellular content, or interstitial fluid was measured with a lactic acid assay kit (Nanjing Jiancheng Bioengineering Institute, China).

### Statistical analysis

Unpaired Student's *t*-test with two tails was used to determine the significance of the differences between the two groups. One-way ANOVA was performed for comparisons among more than two groups with an FDR post hoc analysis.

## Supplementary Material

load041_suppl_Supplementary_Figures_S1-S4

## Data Availability

The RNA-Seq data have been deposited in the NCBI Sequence Read Archive (SRA) database with accession number PRJNA827599. All study data are included in the article and/or Supplementary Materials. Materials are available upon request.
