# Supplementary material for: Intestinal monocarboxylate transporter 1 mediates lactate transport in the gut and regulates metabolic homeostasis of mouse in a sex-dimorphic pattern
Source: Life Metab. 2023 Nov 6;3(1):load041. doi: 10.1093/lifemeta/load041 (PMC11748985; doi:10.1093/lifemeta/load041)
Supplement: load041_suppl_Supplementary_Figures_S1-S4 [file load041_suppl_Supplementary_Figures_S1-S4.pdf]

Supplemental Materials

Detailed Materials and Methods

Mice and diets

Slc16a1$^{fl/fl}$ mice were obtained from the Model Animal Research Center of Nanjing University (Nanjing, China) as previously described [1]. Villin-cre mice were purchased from Shanghai Model Organisms Center (Shanghai, China). All mouse strains are on the C57BL/6 genetic background. For specific deletion of *Slc16a1* gene in intestinal epithelium, *Slc16a1*$^{fl/fl}$ mice were crossed with villin-cre mice to generate *Slc16a1*^{IKO} mice. The mice were genotyped by PCR method using genomic DNA isolated from the tails. Genotyping of the mice expressing *Slc16a1* alleles with floxed site was performed with primers 5′-TGTACCAGCCACCGTCCTT-3′ and 5′-CATCTTGCCTGAGCGTCTAA-3′. Genotyping of mice expressing villin-cre was performed with primers 5′-GCCTTCTCCTCTAGGCTCGT-3′, villin-cre-P2: 5′-TATAGGGCAGAGCTGGAGGA-3′, and 5′-AGGCAAATTTTGGTGTACGG-3′. *Slc16a1*$^{fl/fl}$ (WT) and *Slc16a1*^{IKO} littermates aged 5-6 weeks were fed with normal chow diet or a HFD (D12492, with 60% calories from fat (Research Diets, USA) for 18 weeks. Mice were maintained in specific pathogen-free (SPF) facility and kept on a 12 h light/dark cycle with free access to food and water. All animal experimental protocols were approved by Institutional Animal Care and Use Committee Institutional Animal Care and Use Committee of Shanghai Institute of Nutrition and Health, Chinese Academy of Sciences (CAS) (IACUC, SINH, CAS) with an approval number SINH-2020-CY-1

Body composition and metabolic cage analyses

The body composition of the mice was assessed by echoMRI (Houston, USA). The data of total body fat mass and lean mass were collected for each mouse according to manufacturer's directions. For metabolic cage analysis, the mice were randomly chosen (n = 4 for each group) for determination of metabolic rate using the comprehensive laboratory animal monitoring system (CLAMS-16, Columbus Instruments, USA) according to the manufacturer's instructions. Mice were allowed to acclimate to the system for the first 24 h and the data were collected for the next 48 h. Oxygen uptake (VO_2), carbon dioxide production (VCO_2) and respiratory exchange ratio (RER) were recorded. All the parameters were collected every 16 min. The values of RER were normalized to lean body mass when used for comparison among different groups.

Oral glucose tolerance test (oGTT) and insulin tolerance test (ITT)

For the oGTT, the mice were fasted overnight for 16 h and subsequently given 2 g/kg of D-(+)-glucose solution (Sigma-Aldrich, USA) by oral gavage. For ITT, the mice were fasted for 4 h and subsequently injected intraperitoneally with 0.8 U/kg of insulin solution (Novo Nordisk, Denmark). Blood glucose was determined from tail vein at 0, 15, 30, 60, 90 and 120 min after glucose or insulin administration by using a glucometer (Bayer,

German).

Measurement of plasma and liver parameters

The mice were euthanized and the blood was immediately collected from the heart into EDTA-K2-treated microtubes (Kangjian Medical, China). The blood samples were centrifuged at 1,900 g for 10 min and the supernatant plasma was collected and divided into 3 portions for different usages. All plasma samples were stored at −80 °C and multiple (>2) freeze/thaw cycles were avoided. Hepatic lipids were extracted by a previously reported method [2]. Plasma or hepatic levels of triglycerides (TG), total cholesterol (TC), high-density lipoprotein cholesterol (HDL-C) and low-density lipoprotein cholesterol (LDL-C) were determined by the colorimetric method with corresponding kits (ShenSuo UNF, China) according to manufacturer's instructions.

Analysis of short chain fatty acids (SCFAs)

Mice were pre-treated with inulin (Titan, China) solution (5 g/kg/day) for 7 consecutive days by oral gavage. For the pre-treatment of plasma samples, portal vein blood was collected and 200 μL of plasma was mixed with 800 μL of methanol, followed by thorough vortex and settled by gravity for 1 h. The solution was then centrifuged at 11,000 rpm for 30 min at 4°C. The supernatant was collected and vacuum-concentrated by a vacuum pump. The samples were then centrifuged at 11,000 rpm for 5 min, and the supernatant was stored at -80°C for detection. Determination of short chain fatty acids including acetic, propionic, butyric, and pentanoic acids was performed by gas chromatography (Agilent 7890B Series, Agilent, US) using a capillary DB-FFAP column (30 m, 0.25 mm, 0.25 μm; Agilent, US), coupled to a flame ionization detector (GC-FID). The column temperature was programed at 70°C and held for 6 min, rising to 140°C at 5°C/mined, held for 2 min, elevating to 200°C at 50°C/min, and then held for 3 min (total run time 26.2 min). The carrier gas was helium (1.5 mL/min). The injection was carried out with a split injector (1:2) at 250°C and detector temperature was 300°C. 1 μL of the solution was injected into the GC-FID system. Different SCFAs were identified according to the retention time of corresponding standard compounds (acetic acid, propionic acid, butyric acid, and pentanoic acid; Sigma-Aldrich) and their quantification was determined with reference to the corresponding standard curves.

Intestinal transcriptome analysis via RNA-Seq

For RNA extraction, the mouse jejunum tissues were frozen in liquid nitrogen and stored at -80°C. Total RNA was isolated from these samples using TRIzol Reagent (Invitrogen, US) according to the manufacturer's instructions, and genomic DNA was removed using DNase I (Takara, Japan). RNA quality (RNA integrity number, RIN) was determined by Agilent 2100 Bioanalyzer (Agilent, US). RNA concentration was determined by a NanoDrop 2000 (Thermo Scientific, US) and the integrity was also examined by agarose gel electrophoresis. Only high-quality RNA samples (OD260/280 = 1.8 ~ 2.2, OD260/230

≥ 2.0, RIN ≥ 6.5) were used to construct sequencing library. The transcriptome library was prepared following TruSeq RNA sample preparation Kit from Illumina (Illumina, US) using 1μg of total RNA. Shortly, mRNA was isolated according to polyA selection method by oligo(dT) beads and then fragmented at fragmentation buffer. The double-stranded cDNA was synthesized using a SuperScript double-stranded cDNA synthesis kit (Invitrogen, US) with random hexamer primers (Illumina, US). The synthesized cDNA was then subjected to end-repair, phosphorylation and 'A' base addition according to Illumina's library construction protocol. Libraries were size-selected for cDNA target fragments of 300 bp on 2% Low Range Ultra Agarose (Bio-Rad, US) followed by PCR amplification using Phusion DNA polymerase (NEB, US) for 15 cycles. After quantification by TBS380, paired-end RNA-seq sequencing library was sequenced with the Illumina HiSeq x ten/NovaSeq 6000 sequencer (2 × 150bp read length). The raw paired end reads were trimmed and quality controlled by SeqPrep (https://github.com/jstjohn/SeqPrep) and Sickle (https://github.com/najoshi/sickle) with default parameters. Clean reads were then separately aligned to reference genome of *Mus musculus* (GRCm38.p6) with orientation mode using HISAT2 (http://ccb.jhu.edu/software/hisat2/index.shtml) [3] software. The mapped reads of each sample were assembled by StringTie (https://ccb.jhu.edu/software/stringtie/index.shtml?t=example) in a reference-based approach [4]. The transcriptome data were analyzed on the online platform of Majorbio Cloud Platform (www.majorbio.com) [5]. To identify DETs (differential expression transcripts) between two different groups, the expression level of each transcript was calculated according to the transcripts per million reads (TPM) method. RSEM (http://deweylab.biostat.wisc.edu/rsem/) [6] was used to quantify gene abundances. Essentially, differential expression analysis was performed using the EdgeR [7] with p value < 0.05 and DETs with $|\log_{1.5}FC| \geq 1$ were considered to be significantly different expressed transcripts). In addition, functional-enrichment analysis including GO and KEGG were performed to identify enriched GO terms and metabolic pathways based on the major DETs. To identify the major DETs, the GENCODE Basic transcript set (release M25) was used to be cross-compared with previous DETs. GENCODE Basic transcript dataset was prioritized for full-length protein coding transcripts over partial or non-protein coding transcripts within the same gene [8]. GO functional enrichment and KEGG pathway analysis were carried out on the basis of major DETs by Goatools (https://github.com/tanghaibao/Goatools) and KOBAS (http://kobas.cbi.pku.edu.cn/home.do) [9].

Hematoxylin-eosin staining

Mice tissue samples were dissected and fixed in 4% polyformaldehyde for 48 h and embedded in paraffin. Paraffin-embedded sections (4 μm) were subjected to standard hematoxylin-eosin staining (Servicebio, China). Images were captured using a microscope (Carl Zeiss, Germany).

Immunofluorescence staining

Paraffin-embedded sections (4 μm) of small intestine were used for immunofluorescence staining analysis. The slides were deparaffinized in xylene and rehydrated via ethanol series with gradient (100%, 95%, 80% and 70%) then washed in deionized water. For antigen retrieval, the slides were boiled in 10 mM sodium citrate buffer (pH 6.0) for 30 minutes at 100°C and then maintain at a sub-boiling temperature for 10 minutes. The slides were then incubated with blocking buffer (containing 5% goat serum and 0.1% Triton X-100 in PBS) for 1 h at room temperature and stained with rabbit anti-MCT1 antibody (ABclonal Technology, cat#A3013, China) or rabbit anti-F4/80 antibody (Cell Signaling Technology, cat#30325, US) at 4°C overnight. After washing with PBS, the slides were sequentially incubated with secondary antibody (Alexa Fluor 488 goat anti-rabbit IgG, Life Technologies, cat#A-11008, US) and with Hoechst 33342 (Invitrogen, cat#H3570, US). Slides were washed with PBS, mounted with a mounting medium (Sigma, US), and imaged using a Zeiss LSM880NLO FLIM confocal microscope (Carl Zeiss, Germany).

ELISA

Plasma TNF-α, IL-1β, IL-6, CCL-2 and 17β-estradiol were determined respectively by corresponding ELISA kits (Shanghai Enzyme-linked Biotechnology, China) according to manufacturer's instructions.

Western blotting analysis

Mice tissues were lysed with RIPA lysis buffer (meilunbio, China) with protease and phosphatase inhibitors. Protein concentrations were determined by Enhanced BCA Protein Assay Kit (Beyotime, China). The samples were subjected to sodium dodecyl sulfate-polyacrylamide gel electrophoresis (SDS-PAGE) on 10% running gels, transferred to polyvinylidene fluoride membranes, and incubated with 3% BSA in TBST (Tris-buffered saline, 0.1% Tween 20) at room temperature for 1 h. The membrane was then incubated with primary antibodies against Akt (Cell Signaling Technology, cat#4685, USA), Phospho-Akt (Ser473) (Cell Signaling Technology, cat#4060, US), β-actin (Santa Cruz Biotechnology, cat#sc-47778), or α-tubulin (Sigma, cat#T6074, US) overnight at 4°C. The membranes were then incubated with HRP-conjugated secondary antibodies (Invitrogen, cat#31460, cat#31430) for 1 h at RT, washed and visualized via SuperSignal West Dura Extended Duration Substrate (Thermo Fisher Scientific, US) and western blot imaging system (Tanon, China).

Cell culture

HCT116 cells were cultured in DMEM (Gibco, US) supplemented with 10% fetal bovine serum (FBS) and 1% penicillin-streptomycin. HCT15 cells and RAW264.7 cells were cultured in RPMI-1640 medium (Gibco, US) supplemented with 10% FBS and 1% penicillin-streptomycin. Caco-2 cells were cultured in DMEM supplemented with 20% FBS and 1% penicillin-streptomycin. All cells were cultured at 37 °C in a 5% CO_2

humidified atmosphere. The RAW264.7 macrophages were polarized to M1 inflammatory macrophage by treating with 1 μg/ml LPS (MedChemExpress, US) for 12 hrs.

Estrogen administration

17β-estradiol (E2) (Sigma-Aldrich, US) was dissolved in olive oil. The mice received an intraperitoneal injection of E2 (10 mg/kg of body weight) every other day for 2 weeks.

Generation of antibiotic-induced microbiota-depleted (AIMD) mice

To deplete the intestinal microorganisms, mice were treated with a combination of four non-absorbable antibiotics: ampicillin, neomycin, metronidazole and vancomycin (Sangon Biotech, China) via oral gavage (0.2 mL) for 5 consecutive days (10 mg of each antibiotic per mouse per day) followed by administration in drinking water (ampicillin, neomycin and metronidazole: 1 g/L; vancomycin: 500 mg/L).

Quantitative real-time PCR

Mouse tissues were frozen in liquid nitrogen and stored at -80°C. Total RNA was extracted from tissue samples or cell cultures using TRIzol Reagent (Invitrogen, US). cDNA was synthesized from 1 μg of total RNA using FastKing RT Kit (With gDNase) (TIANGEN Biotech, China). Quantitative real-time PCR (qPCR) primer sequences are included in Supplementary Table 1. The relative expression levels of genes were normalized to their corresponding *Actb* or *Gapdh* levels.

Isolation of interstitial fluid

The extracellular fluid was extracted from mice intestines using a rapid isolation procedure described previously [10]. Briefly, the intestinal tissues were dissected and subjected to centrifugation (800 g, 10 min) at 4°C and then filtered using a 20 μm nylon mesh filter (Millipore, Germany). The extracellular fluid was collected and subsequently used for determination of lactate concentration.

Isolation of intestinal lamina propria lymphocytes (LPLs)

The isolation of LPLs was carried out as previously described [11]. In brief, the large intestines were dissected, and the adipose tissues and Peyer's patches were removed. The intestines were cut open longitudinally and dissected into about 6 pieces. Intestinal pieces were washed with PBS, shaken in PBS containing 1 mM dithiothreitol (DTT) for 10 min at room temperature, and incubated with PBS containing 30 mM ethylenediaminetetraacetic acid (EDTA) at 37°C for 10 min with shaking for 2 cycles. The sections were then digested in RPMI1640 medium (Thermo Fisher Scientific, US) containing DNase I (150ug/ml, Sigma Aldrich, US) and collagenase VIII (200 U/ml, Sigma Aldrich, USA) at 37°C in 5% CO_2 incubator for 1.5 hrs. The digested tissues were homogenized by vigorous shaking and passed through a 100 μm cell strainer (BD biosciences, US). Mononuclear cells were then harvested from the interphase of an 80%

and 40% Percoll (GE Healthcare, US) gradient after a spin at 2500 rpm for 20 min at room temperature.

Flow cytometry

Dead cells were firstly excluded from the analysis with the Fixable Violet Dead Cell Stain Kit (for 405 nm excitation, Thermo Fisher Scientific, US). Antibody staining was performed in PBS containing 2mM EDTA and 0.1% BSA. MHC Class II-FITC (cat#11-5321-82, Thermo Fisher Scientific, USA), CD45-APC-Cyanine7 (cat#A15395, Thermo Fisher Scientific), F4/80-PE (cat#12-4801-82, Thermo Fisher Scientific), and CD11b-PE-Cyanine7 (cat#25-0112-82, Thermo Fisher Scientific) antibodies were used in the experiments. Flow cytometry data were collected using the Gallios flow cytometer (Beckman Coulter, USA) and analyzed with FlowJo software (TreeStar, USA).

Fecal microbiota transplantation (FMT)

The procedure of FMT was performed following a previously-reported protocol [12]. In brief, the mice were treated with a cocktail of ampicillin (1g/L), neomycin (1g/L), metronidazole (1g/L) and vancomycin (0.5 g/L) in the drinking water (Sangon Biotech, China) for 7 consecutive days to remove indigenous gut microorganisms. After a 2-day recovery, mice were received FMT for 3 consecutive days on the first week and once a week for the duration of the experiment. Fresh stool were collected from the donor mice, homogenized at 50 mg/ml in sterile PBS, settled by gravity for 2 min and the supernatant was administrated 200μl to each recipient mouse via oral gavage.

Gut microbiota analysis

For sample collection, fresh fecal samples were collected from *Slc16a1*$^{fl/fl}$ and *Slc16a1*^{IKO} male mice fed normal chow at the age of 20 week. Samples were collected into sterile tubes on ice and stored at -80°C within 1 h. Fecal genomic DNA was extracted using the E.Z.N.A. stool DNA Kit (Omega Bio-tek, US) according to manufacturer's instructions. The quality and concentration of DNA were determined by 1.0% agarose gel electrophoresis and a NanoDrop 2000 spectrophotometer (Thermo Scientific, US). The V3-V4 region of the bacterial 16S rRNA gene was amplified with primer pairs 338F (5'-ACTCCTACGGGAGGCAGCAG-3') and 806R (5'-GGACTACHVGGGTWTCTAAT-3'). For PCR reaction, 4 μL 5 × Fast Pfu buffer, 2 μL 2.5 mM dNTPs, 0.8 μL each primer (5 μM), 0.4 μL Fast Pfu polymerase, 10 ng of template DNA, and ddH_2O were mixed to a final volume of 20 μL. All PCR amplifications were performed in a thermocycler PCR system (ABI GeneAmp 9700, US) following these steps: initial denaturation at 95°C for 3 min, followed by 27 cycles of denaturing at 95°C for 30 s, annealing at 55°C for 30 s and extension at 72°C for 45 s, and final extension at 72°C for 10 min. All samples were amplified in triplicate. The PCR products were extracted from 2% agarose gel and purified using the AxyPrep DNA Gel Extraction Kit (Axygen Biosciences, US) according to manufacturer's instructions and quantified using Quantus Fluorometer (Promega, US). The

purified amplicons were mixed at equal molar ratios and paired-end sequenced on an Illumina MiSeq PE300 platform (Illumina, US) according to the standard protocols by Majorbio Bio-Pharm Technology Co. Ltd. (Majorbio, China). The sequencing data were analyzed on the online platform of Majorbio Cloud Platform (www.majorbio.com) [5]. After demultiplexing, the sequences were quality filtered with fastp (0.19.6) [13] and merged with FLASH (v1.2.11) [14]. Then the high-quality sequences were de-noised using DADA2 [15] in the Qiime2 (v2022.2) [16] pipeline with recommended parameters which obtains single nucleotide resolution based on error profiles within samples. DADA2 denoised sequences are usually called amplicon sequence variants (ASVs). The average number of reads per sample was over 65,000 and the average length per read was 423 bp in our data. To minimize the effects of sequencing depth on α and β diversity analysis, the number of sequences from each sample was rarefied to 41,928, the smallest number of sequences in all samples. Taxonomic assignment of ASVs was performed using the Naive bayes consensus taxonomy classifier implemented in Qiime2 and the SILVA 16S rRNA database (v138). Based on the ASVs information, α diversity analysis including sobs richness index and Shannon index was calculated with Mothur vo1.30.1 [17]. The similarity among the microbial communities in different samples was determined by principal coordinate analysis (PCoA) based on unweighted UniFrac distance using Vegan v2.5-3 package in R v3.3.1. A phylogenetic tree was built using FastTree v2.1.3 (http://www.microbesonline.org/fasttree/). Significant changes in the abundance of genus level between two groups were assessed using the Wilcoxon rank sum test with FDR correction. The linear discriminant analysis (LDA) effect size (LEfSe) [18] (http://huttenhower.sph.harvard.edu/LEfSe) analysis was performed to identify the significantly abundant taxa (phylum to genera) of bacteria between the two groups (LDA score > 3.5, $p < 0.05$).

Lactate measurement

Blood was respectively collected from heart and portal vein. Plasma was obtained by centrifugation at 1,900 g for 10 min. Intracellular content was extracted by lysing cells with repeated freeze-thaw cycles followed by centrifugation at 12,000 g for 5 min. Lactate from samples of plasma, cell media, cellular content or interstitial fluid was measured with a lactic acid assay kit (Nanjing Jiancheng Bioengineering Institute, China) following the manufacturer’s protocol.

Statistical analysis

Data are expressed as mean ± SEM. Unpaired Student’s t-test with two tails were used to determine the significance of the differences between two groups. One-way ANOVA was performed for comparisons among more than two groups with an FDR *post hoc* analysis. To determine if two different factors have an effect on a measured variable, a two-way ANOVA was used, followed by an FDR *post hoc* test. Statistical tests were performed using Microsoft Excel (Microsoft) or Prism 9 (GraphPad Software).

References

1 Lin Y, Bai M, Wang S, Chen L, Li Z, Li C, *et al.* Lactate Is a Key Mediator That Links Obesity to Insulin Resistance via Modulating Cytokine Production From Adipose Tissue. Diabetes 2022;**71**:637-52.

2 Xu D, Wang Z, Zhang Y, Jiang W, Pan Y, Song BL, *et al.* PAQR3 modulates cholesterol homeostasis by anchoring Scap/SREBP complex to the Golgi apparatus. Nat Commun 2015;**6**:8100.

3 Kim D, Langmead B, Salzberg SL. HISAT: a fast spliced aligner with low memory requirements. Nat Methods 2015;**12**:357-60.

4 Pertea M, Pertea GM, Antonescu CM, Chang TC, Mendell JT, Salzberg SL. StringTie enables improved reconstruction of a transcriptome from RNA-seq reads. Nat Biotechnol 2015;**33**:290-5.

5 Ren Y, Yu G, Shi C, Liu L, Guo Q, Han C, *et al.* Majorbio Cloud: A one-stop, comprehensive bioinformatic platform for multiomics analyses. iMeta 2022:e12.

6 Li B, Dewey CN. RSEM: accurate transcript quantification from RNA-Seq data with or without a reference genome. BMC Bioinformatics 2011;**12**:323.

7 Robinson MD, McCarthy DJ, Smyth GK. edgeR: a Bioconductor package for differential expression analysis of digital gene expression data. Bioinformatics 2010;**26**:139-40.

8 Frankish A, Diekhans M, Ferreira AM, Johnson R, Jungreis I, Loveland J, *et al.* GENCODE reference annotation for the human and mouse genomes. Nucleic Acids Res 2019;**47**:D766-D73.

9 Xie C, Mao X, Huang J, Ding Y, Wu J, Dong S, *et al.* KOBAS 2.0: a web server for annotation and identification of enriched pathways and diseases. Nucleic Acids Res 2011;**39**:W316-22.

10 Reddy A, Bozi LHM, Yaghi OK, Mills EL, Xiao H, Nicholson HE, *et al.* pH-Gated Succinate Secretion Regulates Muscle Remodeling in Response to Exercise. Cell 2020;**183**:62-75 e17.

11 Chang J, Ji X, Deng T, Qiu J, Ding Z, Li Z, *et al.* Setd2 determines distinct properties of intestinal ILC3 subsets to regulate intestinal immunity. Cell reports 2022;**38**:110530.

12 Wang S, Huang M, You X, Zhao J, Chen L, Wang L, *et al.* Gut microbiota mediates the anti-obesity effect of calorie restriction in mice. Sci Rep 2018;**8**:13037.

13 Chen S, Zhou Y, Chen Y, Gu J. fastp: an ultra-fast all-in-one FASTQ preprocessor. Bioinformatics 2018;**34**:i884-i90.

14 Magoc T, Salzberg SL. FLASH: fast length adjustment of short reads to improve genome assemblies. Bioinformatics 2011;**27**:2957-63.

15 Callahan BJ, McMurdie PJ, Rosen MJ, Han AW, Johnson AJ, Holmes SP. DADA2: High-resolution sample inference from Illumina amplicon data. Nat Methods 2016;**13**:581-3.

16 Bolyen E, Rideout JR, Dillon MR, Bokulich NA, Abnet CC, Al-Ghalith GA*, et al.* Reproducible, interactive, scalable and extensible microbiome data science using QIIME 2. Nat Biotechnol 2019;**37**:852-7.

17 Schloss PD, Westcott SL, Ryabin T, Hall JR, Hartmann M, Hollister EB*, et al.* Introducing mothur: open-source, platform-independent, community-supported software for describing and comparing microbial communities. Appl Environ Microbiol 2009;**75**:7537-41.

18 Segata N, Izard J, Waldron L, Gevers D, Miropolsky L, Garrett WS*, et al.* Metagenomic biomarker discovery and explanation. Genome Biol 2011;**12**:R60.

Supplemental Figures

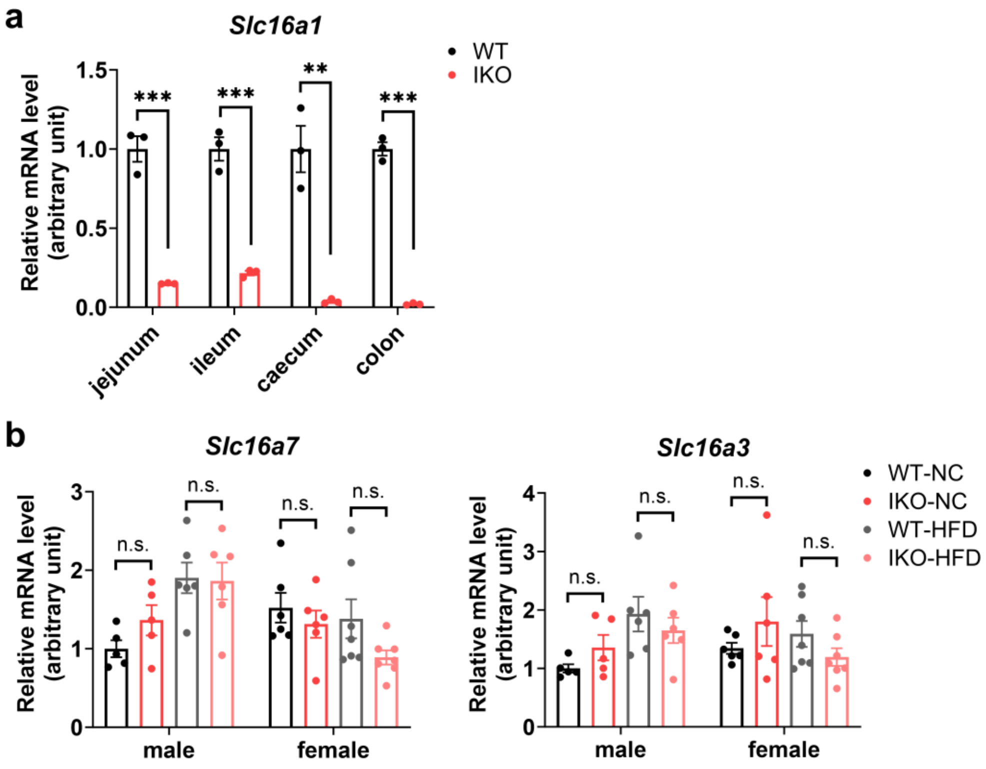

Figure S1. Validation of intestine-specific *Slc16a1* knockout mice.

(a) Relative mRNA expression of *Slc16a1* at different positions of mice intestine. n = 3 for each group. (b) qPCR of *Slc16a7* and *Slc16a3* mRNA levels in WT and *Slc16a1*^{IKO} mice. n = 5-7 for each group. Data are expressed as mean ± SEM. ** p < 0.01. *** p < 0.001.

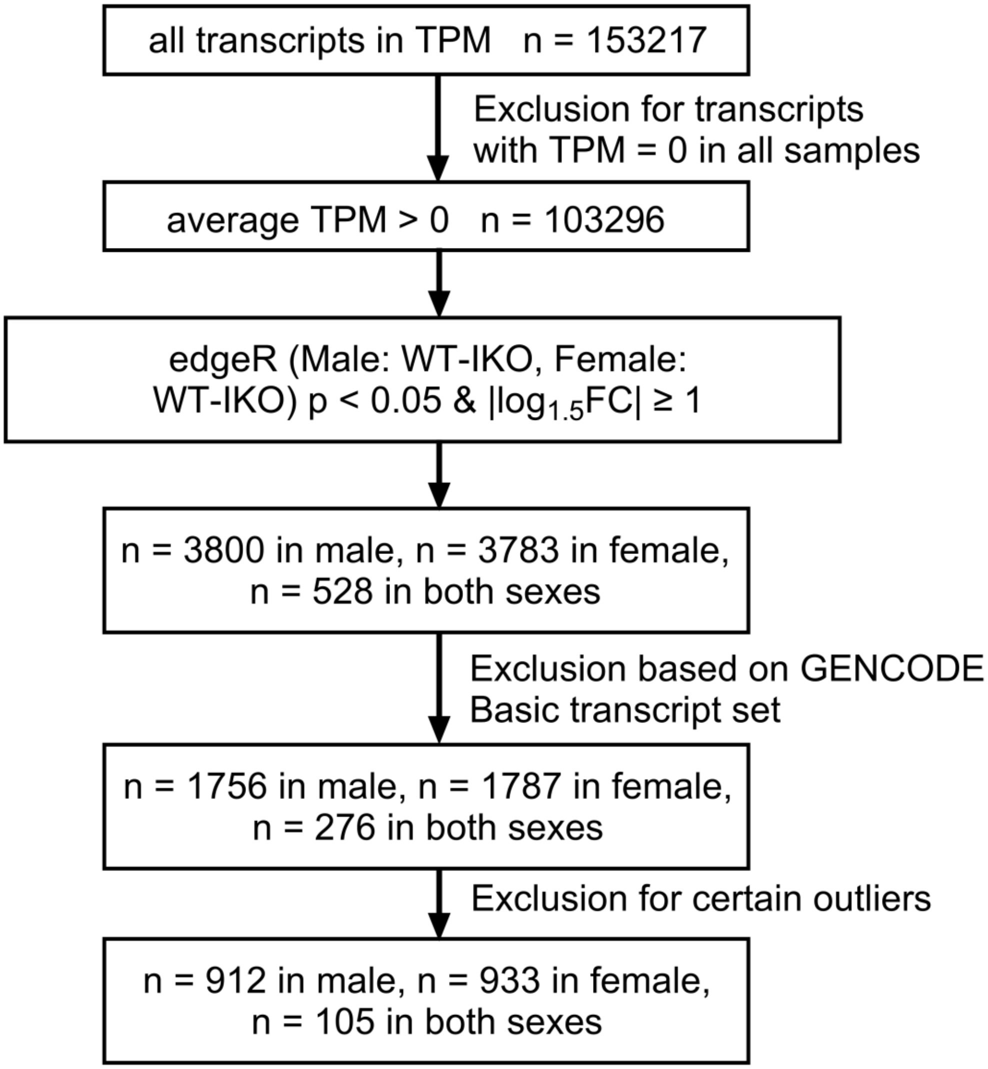

Figure S2. Flow scheme of transcriptome analysis.
Flow scheme of transcriptome analysis with corresponding inclusion and exclusion criteria.

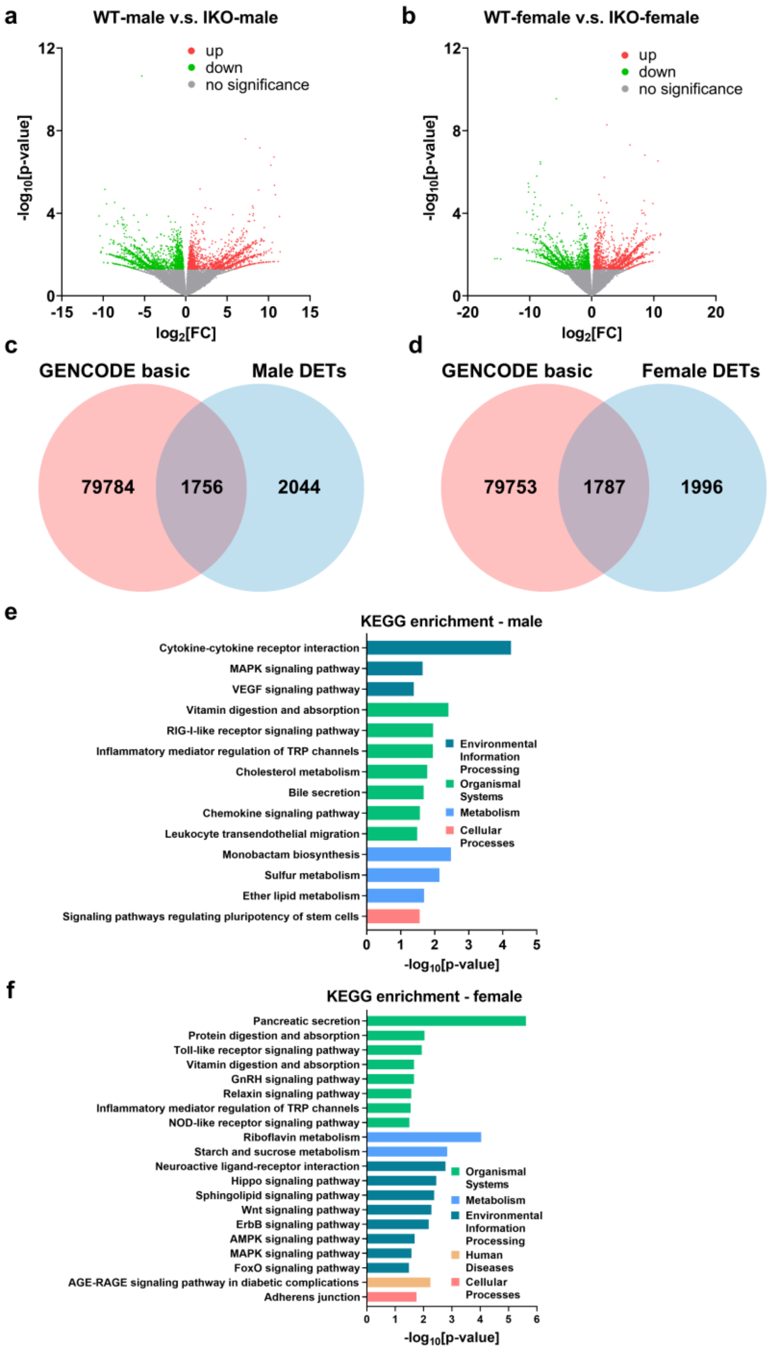

Figure S3. Transcriptome analysis of intestine-specific *Slc16a1* knockout mice.
(a & b) Volcano plots of the sequencing data from the intestines of WT and *Slc16a1*^{IKO} mice. FC, fold change. (c & d) Identification of major DETs in male and female mice illustrated by Venn diagram. (e & f) KEGG enrichment analysis of the major DETs in male (e) and female (f) mice.

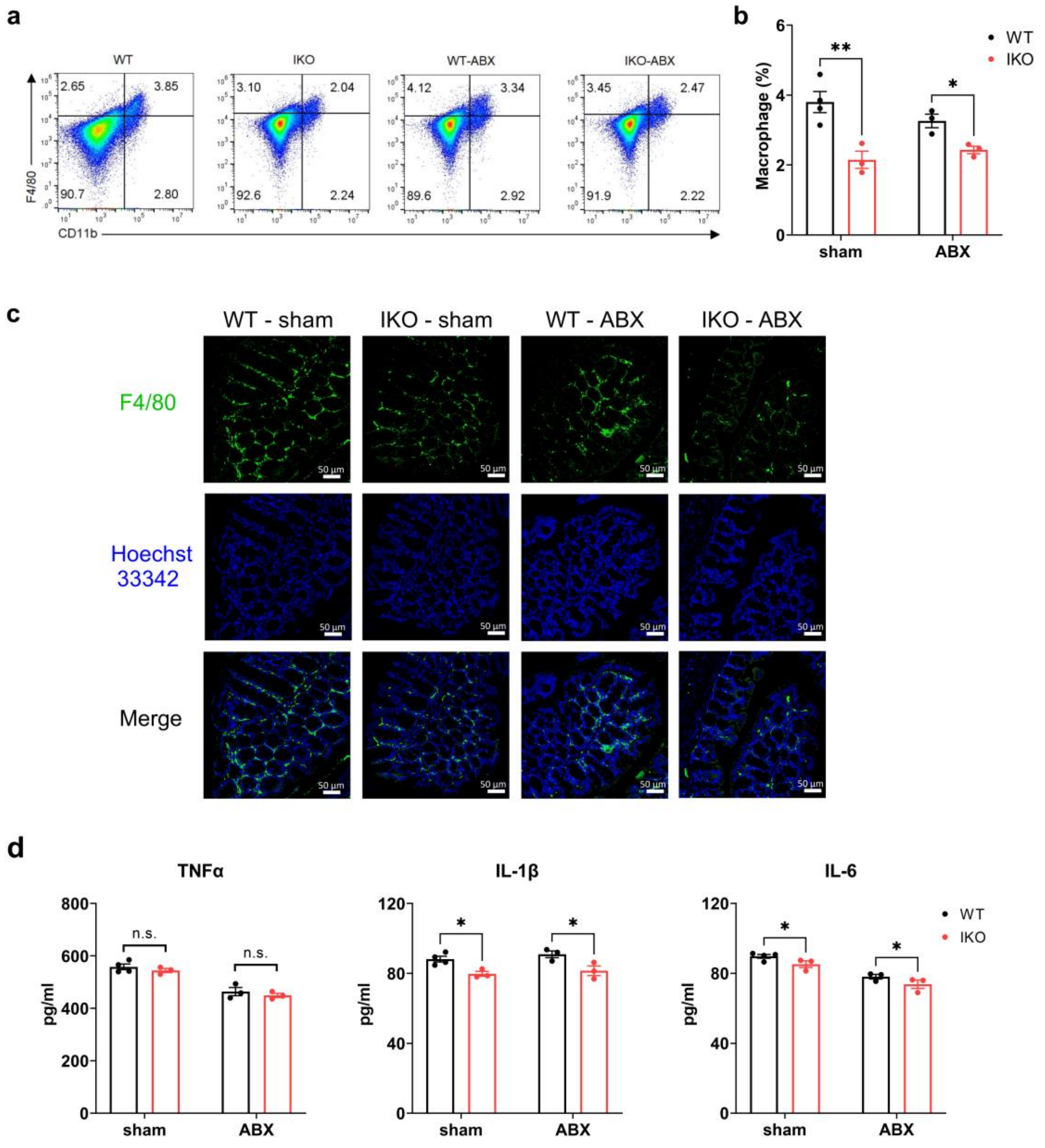

Figure S4. The impacts of antibiotic-induced microbiota depletion on gut macrophages and inflammatory cytokines of male mice

(a and b) Flow cytometry analysis for F4/80^{+}CD11b^{+} macrophages (gated on CD45^{+}MHCII^{+} cells) using isolated intestinal lamina propria lymphocytes (LPLs) in WT and *Slc16a1*^{IKO} male mice with or without antibiotic treatment. n = 3-4 for each group. (c) Immunofluorescence staining of intestinal F4/80 in WT and *Slc16a1*^{IKO} male mice. Scale bar, 50 µm. (d) Circulating levels of TNF-α, IL-1β and IL-6 in WT and *Slc16a1*^{IKO} male mice from AIMD model determined by corresponding ELISA kits. n = 3-4 for each group. * $p < 0.05$. ** $p < 0.01$, n.s. for non-significant.
